# Differences in extinction selectivity and their relationship to functional traits in late Cenozoic mollusks

**DOI:** 10.7717/peerj.20715

**Published:** 2026-03-03

**Authors:** Daniel Rojas-Ariza, Luke C. Strotz, Bruce S. Lieberman

**Affiliations:** 1 Department of Ecology & Evolutionary Biology, University of Kansas, Lawrence, KS, United States of America; 2 The University of Kansas Biodiversity Institute, Lawrence, KS, United States of America; 3 Department of Earth Sciences, Faculty of Geosciences, Utrecht University, Utrecht, Netherlands

**Keywords:** Extinction selectivity, Bivalves, Gastropods, Functional traits, Late Cenozoic

## Abstract

Identifying generalizable patterns of extinction selectivity is crucial for understanding the mechanisms driving extinction processes. Differences in the trait composition of extinct surviving species may represent evidence of processes of extinction selectivity during the past. Here, we leverage the information from the late Cenozoic molluscan fossil record and extant biota from the western Atlantic coast of North America, to test for differences in the trait composition of extinct and surviving species of bivalves and gastropods. We found basal metabolic rate (BMR) is the trait most closely associated with the extinction patterns observed in our data. On average across all studied molluscan species, odds of survival decrease by ∼11% for every 1-milliwatt (mW) increase in BMR, but this pattern was consistent only for bivalves. BMR thus represents an organismal trait that scales up to predict species survival in bivalves. By contrast, a variety of other functional traits shown to be important in other taxonomic and temporal contexts, including shell composition in bivalves and shell structures such as varices and the callus in gastropods, were not found to be associated with survival. This could suggest some of these traits, sometimes posited to represent important organismic adaptations, may not have played a prominent role in long term species survival in Cenozoic bivalves and gastropods. A variety of biotic and abiotic factors may likely determine the extent to which particular organismal traits influence patterns of species survival.

## Introduction

Human-induced climate change is driving biodiversity loss at an accelerating rate, resulting in the disappearance and transformation of ecosystems globally (Thomas et al., 2004; Pereira et al., 2010). Providing key information that contributes to identifying generalizable patterns of extinction selectivity in specific environmental and biological contexts is crucial for developing predictive frameworks, and will partly depend on understanding the drivers of past extinctions and the role of biological and environmental factors on extinction selectivity (Todd et al., 2002; Lockwood, 2008; Rivadeneira & Marquet, 2007; Harnik, 2011; Harnik et al., 2012; Orzechowski et al., 2015; Strotz et al., 2018; Albano et al., 2024; Malanoski et al., 2024; Mondanaro, Dominici & Danise, 2024). The late Cenozoic deposits of the western Atlantic region of North America provide an abundant, and taxonomically well-studied fossil and extant mollusk record, which has made it an excellent and widely used system for studying patterns of extinction (Allmon et al., 1993; Allmon, 2001; Harnik, 2011; Saupe et al., 2014a; Saupe et al., 2015; Strotz et al., 2018; Pimiento et al., 2020). The last ∼3 million years (Ma) interval is particularly relevant not only because it includes many species that still survive today, but also because it encompasses periods for which climatic conditions in the ocean are expected to coincide closely with those anticipated to occur within the next few centuries (Zubakov & Borzenkova, 1988; Burke et al., 2018; Tierney et al., 2020; Dowsett et al., 2021). In particular, the late Cenozoic has been marked by several episodes of global climate change, characterized by oscillations between glacial and interglacial intervals (Lourens & Hilgen, 1997; Lourens et al., 2010). These factors make the late Cenozoic benthic mollusks from the western Atlantic region of North America a promising system to study processes of extinction that may be extrapolated to the present and future scenarios.

A particularly valuable way of assessing extinction patterns is by considering a trait-based approach (McKinney, 1997; McKinney, 2001; Jablonski, 2005; Kiessling, Aberhan & Villier, 2008; Harnik, 2011; Orzechowski et al., 2015; Collins et al., 2018; Reddin et al., 2020; Monarrez, Heim & Payne, 2021; Huang et al., 2023; Kiessling et al., 2025). The concept of functional traits has been widely used to describe the influence of organismal traits on fitness, and consequently on survival (see Violle et al., 2007; Mlambo, 2014; Chichorro et al., 2022). Here, we consider fourteen molluscan traits (see list in Table 1) and assess the potential correlations between these traits and the extinction selectivity patterns of several late Cenozoic species from the western Atlantic. This selection encompasses traits which have been related to organismal environmental tolerances (*e.g.*, life habit: McRoberts & Newton, 1995; Knoll et al., 1996; Widdicombe & Spicer, 2011; Orzechowski et al., 2015, shell composition: Porter, 2010), responsiveness to environmental perturbations (*e.g.*, shell fixation: Stanley, 1972), predation resistance (*e.g.*, shell ornamentation: Hansen et al., 1999; Klompmaker & Kelley, 2015, varices: Webster & Vermeij, 2017, umbilicus: Hansen et al., 1999; Vermeij, 2020) and resource and metabolic demands (feeding: McRoberts & Newton, 1995; Leonard-Pingel, Jackson & O’Dea, 2012, metabolism: Strotz et al., 2018). The proposed functions of these traits possibly reflect key physiological, biological, and ecological features that may have influenced interspecific differential fitness for bivalves and gastropods at particular intervals of the late Cenozoic, however their specific role (and relative impact) remains sometimes ambiguous.

**Table 1:** Trait coding and classification references. ‘PBDB’ stands for The Paleobiology Database and ‘NMITA’ stands for Neogene Marine Biota of Tropical America database. Asterisks (*) indicate reference level categories defined for traits considered in models.

**Trait**	**Coding**	**Coding reference**
Total dataset		
Life habit	Epifaunal	PBDB
	Semi-infaunal	
	Infaunal*	
Shell composition	Aragonite*	PBDB
	Aragonite/Low Mg calcite	
	Low Mg calcite	
Basal Metabolic Rate (BMR)	Continuous variable	Strotz et al. (2018)
Bivalves		
Feeding type	SU: Suspension feeder*	NMITA
	DS: Surface deposit feeder	
	DC: Chemosymbiotic deposit feeder	
Organism/Substrate relationship	ER: Epifaunal recliner	NMITA
	EP: Epifaunal	
	SI: Semi-infaunal	
	IS: Infaunal siphonate*	
	IA: Infaunal asiphonate	
	WN: Nestler on or within hard substrate	
	WB: Borer, nestling in hard substrate	
Mobility	IM: Immobile*	NMITA
	SE: Sedentary	
	MA: Actively mobile	
	SW: Swimming	
Shell fixation	UN: Unattached	NMITA
	BA: Bysally attached	
	CE: Cemented	
Shell ornamentation	Smooth*	Collected in this study following the classification scheme of Hansen et al. (1999)
	Fine	
	Coarse	
Shell sculpture (ridges morphology)	Commarginal	Collected in this study following the classification scheme of Meinhardt & Klingler (1987) and Ubukata (2005)
	Quasi-commarginal	
	Radial	
	Quasi-radial	
	Superimposed	
	Zigzag	
	Oblique	
Gastropods		
Feeding type	CP: Predatory carnivores	NMITA
	CB: Browsing carnivores	
	HM: Herbivores on fine-grained substrates	
	HR: Herbivores on rock, rubble or coral substrates	
	HP: Herbivores on plant or algal substrates	
	SU: Suspension feeder	
Siphonal canal	Absent	Collected in this study following the classification scheme of Vermeij (2007)
	Notch	
	Parallel to coiling axis	
	Dorsally deflected	
Varices	Absent	Collected in this study following the classification scheme of Webster & Vermeij (2017)
	Round	
	Lamellose	
Callus	Absent	Collected in this study following the classification scheme of Vermeij (2022)
	Cassiform	
Umbilicus	Absent	Collected and classified in this study.
	Smooth	
	Spiny	

To provide more detail, multiple hypotheses have been proposed suggesting different functions for these traits and the roles some of them may have played in influencing the evolutionary history of bivalves and gastropods. For instance, it has been suggested that shell composition might have been subject to selective pressures related to changes in temperature and sea-water chemistry during the Phanerozoic (*e.g.*, Harper, Palmer & Alphey, 1997; Carter, Barrera & Tevesz, 1998; Kiessling, Aberhan & Villier, 2008; Porter, 2010; Balthasar & Cusack, 2015), and has also been associated with shell strength (Troncoso et al., 2020) which is thought to influence predation resistance (Cartwright, Checa & Vendrasco, 2024). Traits such as shell fixation (byssal attachment and cementation) in bivalves have also been proposed to be important biological innovations. Stanley (1972) argued that adult byssal fixation in epifaunal bivalves was an adaptive breakthrough that provided physical stability on hard substrates and contributed to the post-Ordovician expansion of epifaunal taxa and the success and diversification of bivalves overall. Another debate concerns the ecological functions of the different bivalve shell ornamentations and ridge morphologies. Hypotheses include their role in burrowing and protection against predation (Stanley, 1969; Stanley, 1970; Stanley, 1981; Seilacher, 1985; Stanley, 1988; Hansen et al., 1999; Klompmaker & Kelley, 2015). However, Seilacher (1973) suggested that some ridge morphologies such as concentric (commarginal) and radial ribbing may not be truly related to burrowing.

Considering gastropods, shell-sculpture morphologies, such as varices and the callus, have been described as antipredatory structures (Vermeij, 2022; Webster & Vermeij, 2017), that could influence survival and thus affect patterns of extinction. Similarly, the siphonal canal (or siphonal indentation), a characteristic trait of some groups of gastropods, has also been hypothesized to have a variety of functions, including an anti-predatory role, by protecting anterior organs in some cases, as well as being associated with sensory organs, and functions, that detect food, enemies, or mates (Lindberg & Ponder, 2001; Vermeij, 2007). Lastly, an anti-predatory function has also been posited for a closed umbilicus which may enhance burrowing ability and provide greater shell strength (Vermeij, 1987), thereby protecting the shell against external attacks (Vermeij, 2017; Vermeij, 2020). Interestingly, Hansen et al. (1999) did not find support for the hypothesis that species with more antipredatory adaptations are more vulnerable to climate-related mass extinctions, when analyzing both bivalves and gastropods.

Life habit has been intensively studied in the context of extinction selectivity for both gastropods and bivalves, and there have been variable findings regarding its relationship with survival (*e.g.*, no selection: Jablonski & Raup, 1995, epifaunal-favored: Jablonski, 2005; Leonard-Pingel, Jackson & O’Dea, 2012, infaunal-favored: Rivadeneira & Marquet, 2007; Aberhan & Kiessling, 2015; climate-dependent life-habit selectivity: Orzechowski et al., 2015). Feeding type has also been linked to extinction selectivity in both clades (*e.g.*, Rhodes & Thayer, 1991; Todd et al., 2002; Leonard-Pingel, Jackson & O’Dea, 2012). For instance, Leonard-Pingel, Jackson & O’Dea (2012) documented an increase in the abundance of deposit-feeding and chemosymbiotic bivalves relative to suspension-feeding taxa in the Caribbean Neogene, associated with the oceanographic effects caused by the closure of the Panamanian Isthmus. Additionally, Todd et al. (2002) found a significant decline in the abundance, but not diversity, of predatory gastropods and suspension-feeding bivalves during the Plio-Pleistocene. More recently, evidence has been found suggesting that species with higher energy demands have been negatively selected over species with lower energy requirements during the late Cenozoic (Strotz et al., 2018).

The aforementioned studies provide varying degrees of evidence supporting or challenging the contributions of these traits to survival-related factors. Several explicitly assess the selectivity of certain traits across different temporal and spatial scales, with a subset of these studies focusing on specific intervals within the late Cenozoic (*e.g.*, Hansen et al., 1999; Todd et al., 2002; Leonard-Pingel, Jackson & O’Dea, 2012). Despite variability in the strength and focus of the evidence, these collective findings are a guide to which selection of traits are the best to evaluate as potential correlates of survivorship over the last ∼3 million years. If an organismal trait truly enhanced fitness and, consequently, survivorship at certain times—or, conversely, was subject to negative selective pressures, leading to the extinction of certain species—we would expect that there would be a signal of selectivity when comparing extinct and surviving taxa. Differences in functional trait composition between extinct and surviving taxa may have provided evidence of how certain organismal traits influence species extinction (Harnik, 2011; Harnik et al., 2012; Orzechowski et al., 2015; Malanoski et al., 2024). These would represent cases when properties of organisms can ‘scale up’ to explain the extinction of entire species (Strotz et al., 2018; Pimiento et al., 2020). There are other instances, however, where group level properties may be more important in influencing patterns of extinction (*e.g.*, Jablonski & Hunt, 2006). Considering how the environment interacts with traits at different hierarchical levels (*e.g.*, organism, population, species *etc.*) can enhance understanding of how different processes influence macroevolutionary patterns (Lieberman, Miller III & Eldredge, 2007; Jablonski, 2008).

Several studies have highlighted the importance of considering patterns of extinction in mollusks, especially bivalves and gastropods (*e.g.*, Stanley & Campbell, 1981; Jablonski & Hunt, 2006; Smith & Roy, 2006; Rivadeneira & Marquet, 2007; Harnik, 2011; Collins et al., 2018; Huang et al., 2023) as they are key components of marine ecosystems, and provide important food sources for humans (MacKenzie Jr et al., 1997; Khan & Liu, 2019; Smaal et al., 2019). In addition, previous works have focused on fossil mollusks because of their diversity, abundance, and the relative completeness of their fossil record (*e.g.*, Kidwell & Flessa, 1995; Allmon, 1989). Here, we leverage information from the fossil record, in conjunction with data from the extant biota, to test for differences in the trait composition of extinct and extant bivalve and gastropod species, aiming to determine which traits most influenced patterns of extinction selectivity in these clades during the late Cenozoic.

## Materials & Methods

### Taxa

We collected trait data for 105 species, 67 bivalves and 38 gastropods, from the modern and late Neogene of the western Atlantic of North America (Tables S1, S2 and S3). No species that originated during the study interval (Pleistocene or Holocene) were considered. Our species selection encompasses 36 families, various ecological characteristics and status (extinct or survived). We only included species that were well represented in museum collections, are well defined taxonomically (with determinations given in the Neogene Atlas of Ancient Life, Hendricks, Stigall & Lieberman, 2015), had available metabolic rate data in Strotz et al. (2018), and ecological data in the Neogene Marine Biota database (NMITA, Budd et al., 2001). These data-availability related factors, plus the exclusion of species that originated within the study interval, resulted in this sample of 105 species from the total 299 considered in Strotz et al. (2018). Thus, the taxa studied do not represent a completely random sample of all species, and potential biases that could arise due to this are discussed below. In terms of the proportion of taxa considered, according to the Neogene Atlas of Ancient Life (Hendricks, Stigall & Lieberman, 2015)—a detailed source on species from the western Atlantic—our sample represents approximately 16.4% of documented species. For the recent fauna, bivalve species considered herein represent about 10% of the 377 extant species documented by Mikkelsen & Bieler (2007). When the Malacolog database (Rosenberg, 2024) is considered, our sample of extant bivalve species represents roughly 3% of the total estimated species for this group, while our gastropod sample constitutes less than 1% of those documented for the entire western Atlantic in the same database. Nevertheless, it should be noted that unresolved issues with taxonomic identifications may inflate current overall species counts.

### Trait data

We collected data for six traits for bivalves—feeding type, mobility, shell fixation, shell ornamentation, ridges morphology, and organism/substrate relationship (see Table 1 and Table S2). For gastropods, we considered five traits—feeding type, siphonal canal, varix, callus, and umbilicus (see Table 1 and Table S3), while for the total dataset we considered three traits shared by both clades—life habit, shell composition, and basal metabolic rate (BMR) (Table S1).

**Figure 1:**
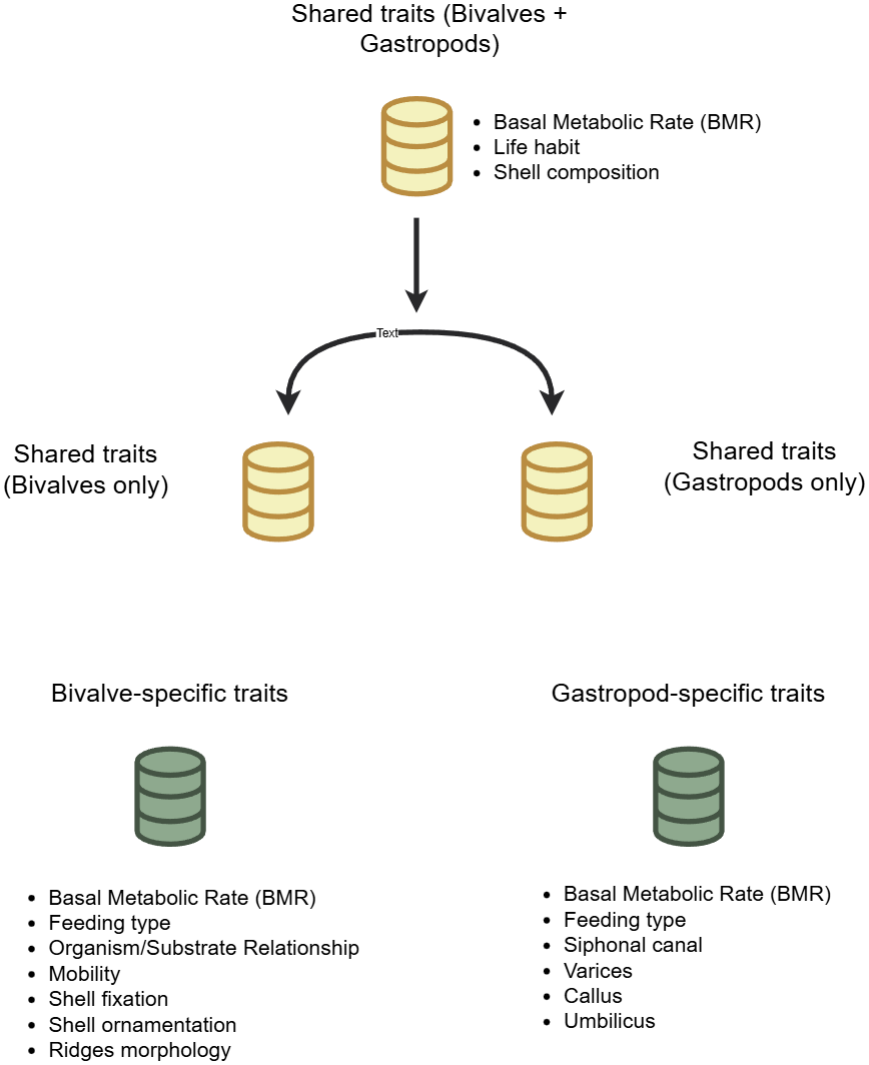
Data structure diagram.

Trait data were obtained from examinations of museum collections, literature, and published databases. All traits were organized into three main datasets for analysis (Fig. 1). A first dataset containing data for traits shared by both clades (Table 1), and two remaining datasets containing clade-specific trait data for each group (Tables S2 and S3). BMR values (measured in watts (W)) for these species were obtained from Strotz et al. (2018), who estimated this trait following Gillooly et al. (2001), based on shell biomass and temperature data. Strotz et al. (2018) estimated shell biomass from maximum linear dimension measurements (L) as a proxy for body size, using two different scaling equations. For bivalves, shell biomass was determined using the equation 1.0 × 10^−5^ × L^2.95^, as described by Payne et al. (2014), while for gastropods, it was estimated using the equation 6.95 × 10^−5^ × L^1.905^, developed by Strotz et al. (2018) based on data from Eklöf et al. (2017). For BMR calculations, we used the temperature data generated by Saupe et al. (2014a) and archived in the corresponding Dryad dataset (Saupe et al., 2014b). These estimations were derived from the coupled atmosphere-ocean HadCM3 general circulation model under four boundary-condition scenarios: Mid-Pliocene Warm Period (mPWP, ∼3.264–3.025 Ma), Last Interglacial (LIG, ∼130–123 ka), Last Glacial Maximum (LGM, ∼21 ka), and the pre-industrial interval (PI, ∼1,850–1,890). For each scenario, the model was first run to equilibrium and then extended for 500 additional years; maximum, minimum, and mean annual temperatures were calculated as 30-year averages from the final portion of each simulation. The geographic coordinates of each measured specimen for a given species were overlaid on the corresponding temperature map for its time slice to estimate the ambient temperature experienced during life. Life habit and shell composition data were obtained from the Paleobiology Database (PBDB, accessed May 2023: Sánchez Roig, 1926; Rudman, 1971; Voigt, 1973; Abbott & Dance, 1986; Todd, 2001; Aberhan et al., 2004; Kiessling, 2004; Mikkelsen & Bieler, 2007; Hendy et al., 2009, see references for each species in Table S1), and when species-level data were not available, the data available for the corresponding genus was used instead. It has been found that certain shell structures of oysters (family: Ostreidae) contain small proportions of aragonite although their shell is mostly calcitic (Stenzel, 1964; Checa, Harper & González-Segura, 2018). For this reason, shell composition data for species within the family Ostreidae specifically were defined based on information from Checa, Harper & González-Segura (2018), Hautmann (2006) and Stenzel (1964). This data does not agree with the information in PBDB, which considers this group to be exclusively calcitic. Therefore, we assessed whether differences in our results were produced between both sources of data (see Tables S1 and S4). Genus-level data on organism/substrate relationship, mobility, and shell fixation for bivalves, and feeding type data for both bivalves and gastropods, were obtained from the NMITA database (https://nmita.rsmas.miami.edu/, Budd et al., 2001). When genus-level data were not available, data were selected from other genera within the same family believed to have similar traits (see “Coding of Citations”—category B in NMITA website). When no description for a particular category is provided in this database (*e.g.*, “HD” feeding type in gastropods), or it was not possible to define the trait state, we classified the trait as “undetermined” (see Trait data caveats in Supplementary Materials) and it was treated as an additional category. Information on ridges morphology and shell ornamentation of bivalves were collected by examining specimens in the collections of the Florida Museum of Natural History (FLMNH; Gainesville, FL, USA) and the Paleontological Research Institution (PRI; Ithaca, NY, USA). Shell sculpture was coded using the classifications proposed by Meinhardt & Klingler (1987) and Ubukata (2005), while shell ornamentation was coded using categories proposed by Hansen et al. (1999). Full details of trait coding and sources used for classification are provided in Table 1. The condition of the siphonal canal, the varix, the umbilicus, and the presence of a callus in gastropods were collected by examining specimens in the FLMNH and PRI, and were coded using the classification schemes listed in Table 1. The presence of an umbilicus was specifically classified in this study as: (1) absent—the umbilicus is closed (*i.e.,* filled with a callus deposit or by an extension of the inner lip as described by Vermeij (2017); (2) smooth—a round umbilicus with a smooth surface and no secondary structures (*e.g.*, spines) along its circumference; or (3) spiny—an umbilicus with spines along its circumference.

Note that although shell ornamentation was implicitly considered for both groups, given that gastropods display a more complex combination of ornamental morphologies than bivalves, and these different morphologies do not necessarily perform the same functions, we considered these traits should be evaluated separately (*e.g.*, varices, callus, and umbilicus). Although both bivalves and gastropods represent ornamental traits, homogenizing them into a few categories would be difficult and introduce uncertainty into the definitions of traits. In addition, some clade-specific trait data obtained from the NMITA database were available only for one of the clades and not both—for instance, mobility was only available for bivalves and not gastropods. Even though mobility data is available for both groups in PBDB (in a more general form), the level of taxonomic detail of that database compared to NMITA is much lower. Further, the trait data from PBDB for the particular taxa studied is not as thoroughly vetted as NMITA, which comprises a more specialized database. For this reason, we decided not to mix data from both sources as this could introduce uncertainty. Thus, in such instances we only used what was available in NMITA, even if it was not available for both groups.

### Analyses

To assess for differences between the trait composition of extinct and surviving taxa, we conducted generalized mixed effects logistic regression models (GLMMs) in R using the mixed() function of the *afex* package (Singmann et al., 2024), where the status of the species was defined as the dependent binary response variable, coded as ‘0’ for extinct or ‘1’ for extant taxa, hence modeled for probability of survival. All analyses were performed in R (R Core Team, 2024). These types of models are particularly powerful to account for hierarchical structures in data. In the case of our data, they incorporate phylogenetic hierarchical structures specifying clades (Bivalvia and Gastropoda) as random effect in the models for the total dataset and family as random effect when clades were analyzed in isolation. By accounting for unexplained variability associated with differences between these taxonomic levels, GLMMs allow us to separate this variability from the fixed effects. This approach provides less biased and more robust estimates of the fixed effects by properly modeling within-group correlations and reducing the risk of overestimating predictor effects. We allowed the intercept to vary while keeping the slope constant in models for each level of the random effects. Moreover, these models are robust to unbalanced designs (Pinheiro & Bates, 1995; Schielzeth et al., 2020), as is the case in this study because of the different number of bivalve and gastropod species considered.

Log-odds ratios were calculated for all parameters in each model, and the 95% confidence intervals (CI) were computed. Odds ratios were derived by calculating the exponential of log-odds ratios (${\mathrm{e}}^{\ln \left( \mathrm{OR} \right) }=\mathrm{OR}$, see formula and explanation in Nick & Campbell, 2007: p. 282; Orzechowski et al., 2015: p. 3598). These ratios are particularly useful as they provide a direct measure of the contribution of each trait (predictor) to extinction/survival (binary outcome), which is why they are helpful in studies addressing extinction selectivity (*e.g.*, Payne et al., 2011; Orzechowski et al., 2015). The second-order Akaike Information Criterion for small sample sizes (AICc) was used for model selection since the ratio of our number of observations and model predictors was <40 (Burnham & Anderson, 2002). We implemented it using the AICc() function of the *AICcmodavg* package (Mazerolle, 2023) in R. Models with a ΔAICc equal to or less than 2 were selected as the best candidates for analysis (Burnham & Anderson, 2002).

We computed 95% CIs of effect sizes of best-supported models *via* parametric bootstrap using the confint() function of the *stats* package (R Core Team, 2024), while bootsrapped *p*-values were computed for these best-supported models *via* type III analysis of variance (ANOVA) tests using the same function used to generate our models. These intervals reduce the probability of Type I error and are typically used in paleontological studies (*e.g.*, Harnik, 2011; Malanoski et al., 2024, however see Arnold, 2010 and Sutherland et al., 2023 for alternative selection criteria).

For the shared traits datasets, we tested for the effect of the additive terms of the three shared traits plus the square term of BMR (BMR^2^), which allowed us to evaluate potential intermediate optimum BMR values (*i.e.,* a parabolic relationship) and to assess whether survival odds decrease towards both extremes (low and high BMR). No interactions involving BMR^2^ were tested as the purpose of considering this term was specifically to test for nonlinear effects of BMR (see Nick & Campbell, 2007: p. 289), and to determine whether survival is maximized at intermediate BMR values. Further, we did not consider interaction-only models (*i.e.,* models that considered only interaction terms without including the main effects of at least one of the interacting predictors) and models that included interactions involving BMR without its main effect. These models do not allow for meaningful interpretation since main effects are essential for understanding interactions. For categorical variables, a reference category was set against which all the other categories were compared to estimate odds ratios (for detailed explanations about reference levels in logistic regression see Nick & Campbell, 2007; Sperandei, 2014). Life habit was coded as a categorical variable with the following levels: infaunal (reference level); semi-infaunal; boring; and epifaunal. Shell composition was also considered as a categorical variable with the following levels: aragonite (reference level), low Mg calcite and both (aragonite/low Mg calcite). Reference levels for clade-specific traits used in models are indicated in Table 1.

Since the original BMR values from Strotz et al. (2018) were measured in watts, the default log-odds ratios for BMR-related coefficients represented the change in the odds of survival for every one W increase or decrease in BMR. However, BMR values in our shared traits dataset range between the order of 1 × 10^−6^ and 1 × 10^−2^W, meaning a one W change is far beyond the scale of natural variation and does not capture meaningful shifts in survival odds. To improve interpretability, we converted BMR values to milliwatts (mW), so that log-odds ratios reflect the change in survival odds for every one mW increase or decrease in BMR. For gastropods specifically, BMR values range between the order of 1 × 10^−6^ to 1 × 10^−3^W. Thus, a 1mW unit of change would still be excessively large and unrealistic, for the same reasons described above. To address this, we rescaled the BMR data by dividing each measurement by the interquartile range of the data, *i.e.,* the difference between the 75th and the 25th percentiles of the data, equal to 7 × 10^−5^W. This approach is equivalent to the procedure recommended by Nick & Campbell (2007: p. 287) for non-normally distributed predictors, as it effectively captures the spread of the middle 50% of the data.

Categorical predictors can present challenges in logistic regression, particularly when some levels have few observations, which can inflate uncertainty, cause quasi-complete separation, or lead to convergence issues (Allison, 2008). In contrast, continuous predictors allow the model to estimate a single trend across all observations rather than separate effects for each level, which generally produces more stable estimates and reduces the risk of fitting problems. To evaluate whether differences in model performance that could arise from using continuous *versus* categorical predictors could impact our results, we conducted sensitivity tests by treating BMR as a categorical variable and compared the results with those obtained using its original continuous form. BMR was categorized using the 25th and 75th percentiles of the distribution of the data. Values lower than the 25th percentile were classified as “low”, values between the 25th and 75th percentiles were categorized as “medium”, and those greater than the 75th percentile were treated as “high” (see Table S1).

To address potential biases in our results due to unbalanced representation of bivalves compared to gastropods, we assessed whether our fixed-effect estimates were consistent with those expected under a balanced dataset. This was done for statistically significant predictors in the best-supported models. We generated 100 balanced subsets by randomly subsampling bivalve species (with replacement) from the original pool of 67 taxa to match the number of gastropod species (*n* = 38). For each subset, we re-estimated the log-odds ratio of predictors. The distribution of estimates from these balanced resamples was then compared to the estimate from the original dataset.

To streamline our analysis and avoid including uninformative parameters (Arnold, 2010; Sutherland et al., 2023), we developed models for the individual clade datasets using traits that initially showed evidence of association with survivorship. This approach aligns with the recommendation from Burnham & Anderson (2002) to construct small sets of models representing plausible research hypotheses that promote valid interpretations. In the case of categorical traits, this initial test of association was done *via* chi-squared tests of independence. We tested for associations between each of the categorical predictors and survival (one at a time). Only those categorical traits that showed a statistically significant association with survival through these chi-squared tests of independence were selected as predictors to be tested in logistic regression models. We followed the Benjamin-Hochberg procedure (Benjamini & Hochberg, 1995) to control for false discovery rate (FDR) and avoid false negatives. The false discovery rate was set to 0.1 following McDonald (2014). Methods controlling for FDR have a greater power (*i.e.,* have higher probabilities of identifying true positives, Chen, Feng & Yi, 2017; Dudoit & Van der Laan, 2008) than methods that control for family wise error rates (FWER), such as the Bonferroni correction. The Benjamin-Hochberg procedure makes sure to identify true positives while allowing a certain proportion of false positives, which we consider to be appropriate in exploratory stages before any confirmatory analyses. BMR was included in all logistic regression models and no initial test for association was performed for this trait in particular given the findings from Strotz et al. (2018).

We additionally investigated whether the higher resolution of trait classification, or the presence of outlier categories (*i.e.,* categories with small sample sizes relative to the others) in certain traits, might impair the ability of our models to detect specific signals in the shared-traits and class-specific datasets. To do this, we examined whether differences in our results would emerge if species were grouped into broader categories for certain traits, particularly those where similar categories could be consolidated and where species exhibited only one state of the trait at a time (species showing more than one state of a trait were not included in these groups). In the case of the shared-traits dataset, we conducted our analyses treating both boring (bivalve species, *n* = 2) and semi-infaunal taxa (*n* = 13) as a single group. For the class-specific traits, we conducted the analyses described above on alternative versions of these datasets for both bivalves and gastropods (Tables S5 and S6, respectively), where the number of categories of bivalve feeding type, ridges morphology, and gastropod feeding type were reduced specifically in cases where certain categories only had a few entries, to avoid potential issues that could be derived from over-splitting categories. In this modified version of the dataset, ridges classified as “quasi-commarginal” in bivalves were included in the “commarginal” category, and “quasi-radial” ridges were considered as “radial”. In addition, bivalve surface deposit feeders (labeled as DS in Table S2) and chemosymbiotic deposit feeders (DC in Table S2) were grouped into one category of deposit feeders (DF in Table S4). In gastropods, predatory and browsing carnivores (CP and CB respectively in Table S3) were clumped into a single category of carnivores (C in Table S5). Similarly, herbivores on fine-grained substrates (HM) and herbivores on plant or algal substrates (HP) were grouped into a single category of herbivores (H in Table S5). Results of this alternative analysis are evaluated and discussed below.

Models that failed to converge (*i.e.,* generated errors when attempting to fit the data) were discarded, while those that successfully produced an output were subjected to further evaluation and diagnostic checks (see Table S7). Common errors encountered when fitting some models include: (1) failures during covariance matrix estimation, often caused by high collinearity among predictors, strong correlations among random effects, or an overly complex random-effects structure relative to the data; and (2) failures during the optimization process, when the algorithm could not find parameter values that sufficiently reduce the deviance, which may result from excessive model complexity, perfect prediction of the response by certain predictors, or very small sample sizes per random-effect level. A series of tests and criteria were used to assess model fit. From the set of models that converged successfully, we identified singular fits—models exhibiting redundant or overparameterized random-effects structures relative to the data—using the isSingular() function of the *lme4* package (Bates et al., 2015) and subsequently excluded them from further analysis. Further, variance inflation factors (VIFs) were calculated using the check_collinearity() function of the *performance* package (Lüdecke et al., 2021) for each of the predictors of the best-supported models to check for multicollinearity. Models for which predictors showed VIFs <3 were retained (Zuur, Ieno & Elphick, 2010). Additionally, we used the functions simulateResiduals() and testResiduals() from the *DHARMa* package (Hartig, 2024) to diagnose model fit and check whether these best models met the distributional assumptions of residuals of GLMMs. Finally, we used the model.avg() function from the *MuMIn* package (Bartoń, 2024) to compute model-averaged coefficients and their corresponding confidence intervals for the cases where multiple best-performing models were retained after applying the selection criteria. These model-averaged coefficients were weighted by the relative support of each best-supported model as determined by AICc scores. When a predictor was absent from a model, a coefficient of zero was assumed during model averaging, thereby incorporating both within- and between-model uncertainty.

## Results

### Extinction selectivity on shared traits: bivalves and gastropods

A total of 154 models were developed: 69 models for the shared-traits dataset (bivalves + gastropods); 69 models when only bivalves were considered; and 11 models when gastropods were considered in isolation. After model selection criteria were applied, two best-supported models were retained when both groups were analyzed in the dataset, three were retained for bivalves (Table 2). When we considered only gastropods in this shared-traits dataset, no models fulfilled our selection criteria, as ten failed to converge and one was singular.

**Figure 2:**
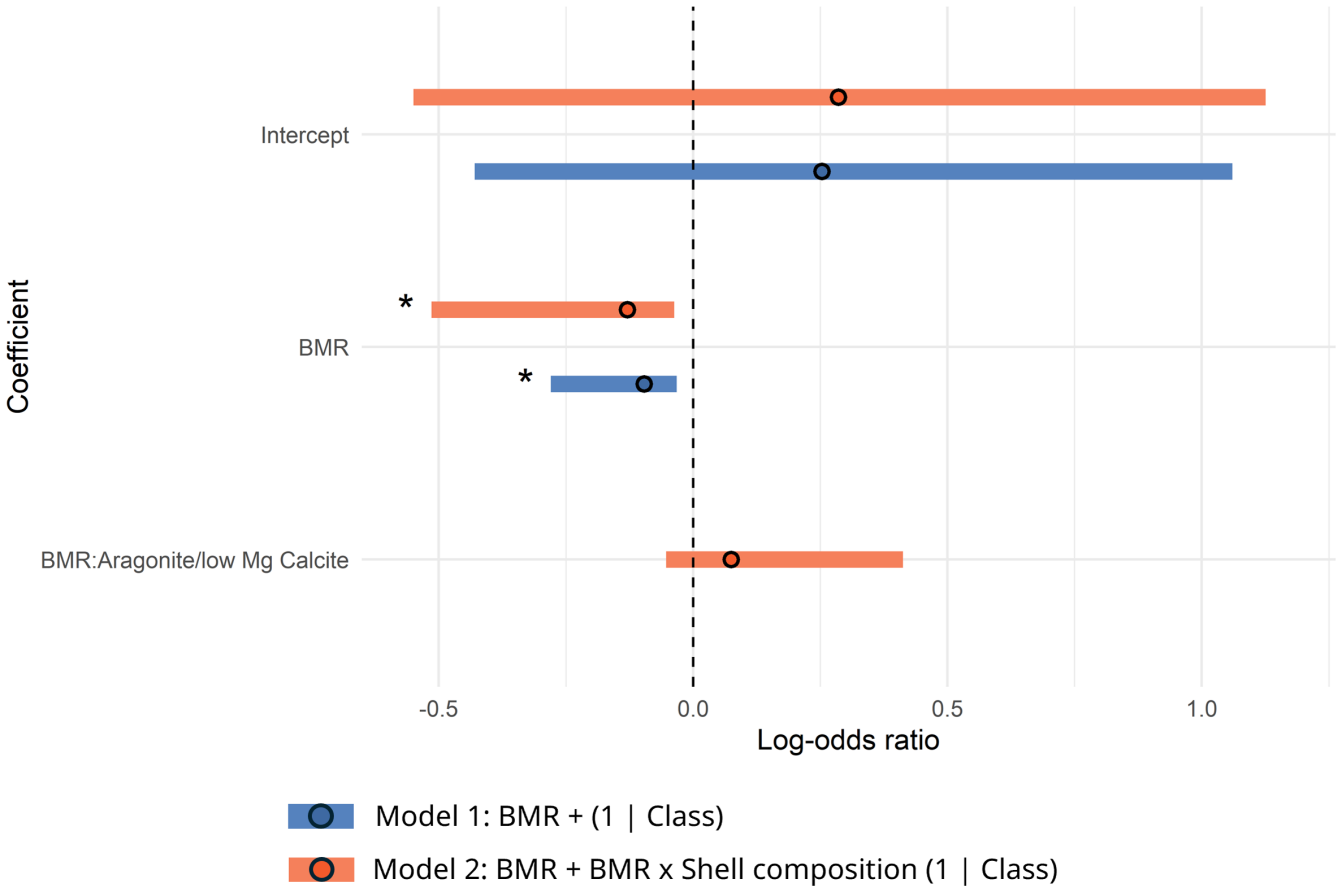
Log-odds ratios Forest plot by model (Shared traits—bivalves and gastropods). The horizontal bars represent 95% confidence intervals. The intercept of the models represents the log-odds of survival when BMR is equal to 0 and the categorical traits of each model are set to their reference level (if the model considers any). The asterisk highlights effect sizes for which their confidence intervals do not overlap 0. Positive values indicate a proportional relationship with survival, and negative values indicate an inverse relationship with survival.

**Figure 3:**
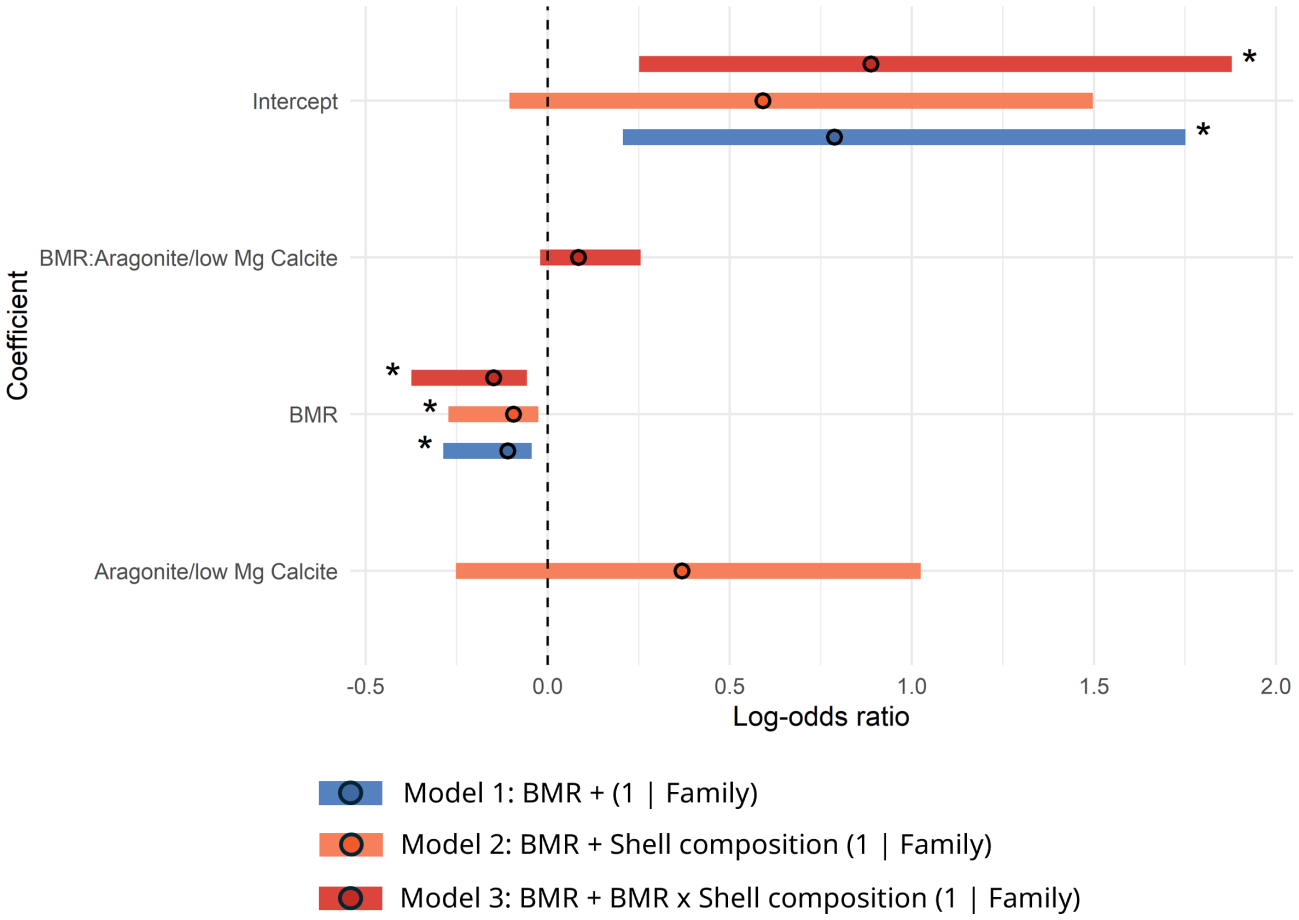
Log-odds ratios forest plot by model (shared traits–bivalves only). The horizontal bars represent 95% confidence intervals. The intercept of the models represents the log-odds of survival when BMR is equal to 0 and the categorical traits of each model are set to their reference level (if the model considers any). The asterisk highlights effect sizes for which their confidence intervals do not overlap 0. Positive values indicate a proportional relationship with survival, and negative values indicate an inverse relationship with survival.

Log-odds ratios estimated from these models suggest a statistically significant effect of BMR on survival (Table 3). Further, on average, between the two models for the shared traits dataset, odds of survival decrease by ∼11% for every 1mW increase in BMR (model-averaged log-odds ratio: −0.115, CI [−0.229, −0.001]; see models in Fig. 2; Table 3). This result was robust to differences in number of bivalve and gastropod species considered (Figs. S1 and S2). An interaction of BMR with shell composition was considered by model 2, however, the effect size of this interaction term in this model is not statistically significant (Table 3). When considering only bivalves in the shared-traits dataset, three best-supported models resulted from the selection process, and we found consistent evidence of the effect of BMR on survival. Again, on average, across models, odds of survival decrease, this time by ∼12% for every 1mW increase in BMR (model-averaged log-odds ratio: −0.125, CI [−0.247, −0.003]; see models in Fig. 3; Table 3). The main effect of BMR on survival is statistically significant in all three best-supported models (Table 3). When considering only gastropods, however, no models met our selection criteria.

**Table 2:** List of best-supported logistic regression models, formulas and corrected Akaike Information Criterion scores (AICc). Interaction terms are indicated by an ‘x’, ‘BMR’ stands for basal metabolic rate, and ‘1 —Class’ or ‘1— Family’ denotes the random intercepts for biological class or family, respectively.

**Dataset**	**Model**	**Formula**	**AICc score**
Shared traits	1	BMR + (1 — Class)	144.9
	2	BMR + BMR x Shell composition + (1 — Class)	144.2
Shared traits –Bivalves only	1	BMR + (1 — Family)	89.0
	2	BMR + Shell composition + (1 — Family)	89.8
	3	BMR + BMR x Shell composition + (1 — Family)	88.1
Bivalves-specific traits	1	BMR + (1 — Family)	89.0
	2	Shell ornamentation + (1 — Family)	87.7

**Table 3:** Best-supported models per dataset. Chi-squared statistics and *p*-values for each trait were obtained from type III ANOVA tests performed on logistic regression models. Log-odds ratios are provided for each trait and for the intercept of each model. Intercepts represent the log-odds of survival when BMR is equal to 0 and/or the categorical traits of a model are set to their reference level. Interaction terms are indicated with an ‘x’, the shell composition reference level used was: ‘aragonite’, and the shell ornamentation reference level used was: ‘smooth ornament’. *P*-values of statistically significant estimates (*p* < 0.05) are highlighted in bold.

**Model**	**Trait**	**Chi-squared statistic**	***P*-value**	**Logistic regression predictor**	**Log-odds ratio**
**Shared traits**					
Model 1	–	–	–	Intercept	0.253
	BMR	6.84	**0.02**	BMR	−0.097
Model 2	–	–	–	Intercept	0.285
	BMR	6.96	**0.01**	BMR	−0.129
	BMR × Shell composition	2.80	0.12	BMR × Shell composition –Aragonite/low Mg calcite	0.075
**Shared traits –Bivalves only**					
Model 1	–	–	–	Intercept	0.787
	BMR	8.63	**0.00**	BMR	−0.110
Model 2	–	–	–	Intercept	0.591
	BMR	6.13	**0.02**	BMR	−0.094
	Shell composition	1.52	0.20	Shell composition—Aragonite/low Mg calcite	0.369
Model 3	–	–	–	Intercept	0.887
	BMR	9.06	**0.00**	BMR	−0.149
	BMR × Shell composition	3.19	0.78	BMR × Shell composition –Aragonite/low Mg calcite	0.085
**Bivalves-specific traits**					
Model 1	–	–	–	Intercept	0.787
	BMR	8.63	**0.00**	BMR	−0.110
Model 2	–	–	–	Intercept	0.673
	Shell ornamentation	12.22	**0.00**	Shell ornamentation –fine ornament	1.882
				Shell ornamentation –coarse ornament	−0.383

Unlike BMR, shell composition was not found to be a significant predictor in any of the best-performing models for the shared traits dataset (Figs. 2 and 3; Table 3). Furthermore, none of the models including life habit as a predictor met our diagnostic criteria, and most exhibited numerical instability or convergence issues, limiting our ability to reliably assess its effect on survival.

When BMR was treated as a categorical predictor, only the previously best-supported models evaluating this trait as the sole predictor for both the shared-traits dataset and the bivalve dataset were retained, and the effect of BMR remained statistically significant (see Table S8). Again, no models passed our diagnostic criteria when gastropods were analyzed in isolation in this case.

When three levels of shell composition were evaluated—treating oyster shells (family Ostreidae, *n* = 4) as exclusively calcitic (low-Mg calcite) rather than both calcitic and aragonitic—no models considering this trait passed our diagnostic criteria. Only the model with BMR as the sole predictor from the original set of best-supported models was retained, both for the combined dataset and for bivalves analyzed alone (Table S9). None of the models considering shell composition converged due to the two main reasons described in the Methods section, limiting our ability to reliably evaluate their effects on survival.

In cases where some levels of a categorical predictor contain few observations, model performance may be affected, producing unstable and highly uncertain effect sizes, and, when some predictor levels perfectly predict the response, preventing model convergence. Given we considered two boring taxa in the shared traits dataset, and both are surviving species, this level perfectly predicts the response and therefore could affect model convergence in some cases. To address the effect of this issue on our models, we conducted our analyses grouping both boring (*n* = 2) and semi-infaunal taxa (*n* = 13). In this case, nine models converged when analyzing both groups, including three that evaluated the main effect of this trait (Table S10). However, these three models did not pass subsequent singularity diagnostics (*i.e.,* they were overfitted in relation to the random-effects structure). Three of the six remaining models passed these previous diagnostics and the AICc selection criteria and included interaction terms between BMR and either life habit or shell composition (models 6 and 7 in ‘Shared traits’, respectively; see Table S10), or both (model 9; Table S10). The main effect of BMR on survival remained statistically significant across all models, while none of the interaction terms were significant. Results remained unchanged when bivalves were analyzed in isolation. A similar pattern was observed when analyzing bivalves in isolation within this shared-traits dataset (see Table S10).

### Clade-specific traits and extinction selectivity

We did not find strong evidence of a link between most clade-specific traits and survival. For the bivalves-specific trait dataset, we found initial evidence for a statistically significant association between survival and three traits: organism/substrate relationship; mobility; and shell ornamentation (rows in bold in Table S11). Then, using this initial evidence, models were analyzed considering these three traits, as well as BMR, which had previously been posited by Strotz et al. (2018) to play an important role in extinction selectivity for molluscan taxa in Pliocene to recent bivalves and gastropods from the western Atlantic. A total of 809 models were tested for the bivalve-specific traits dataset.

**Figure 4:**
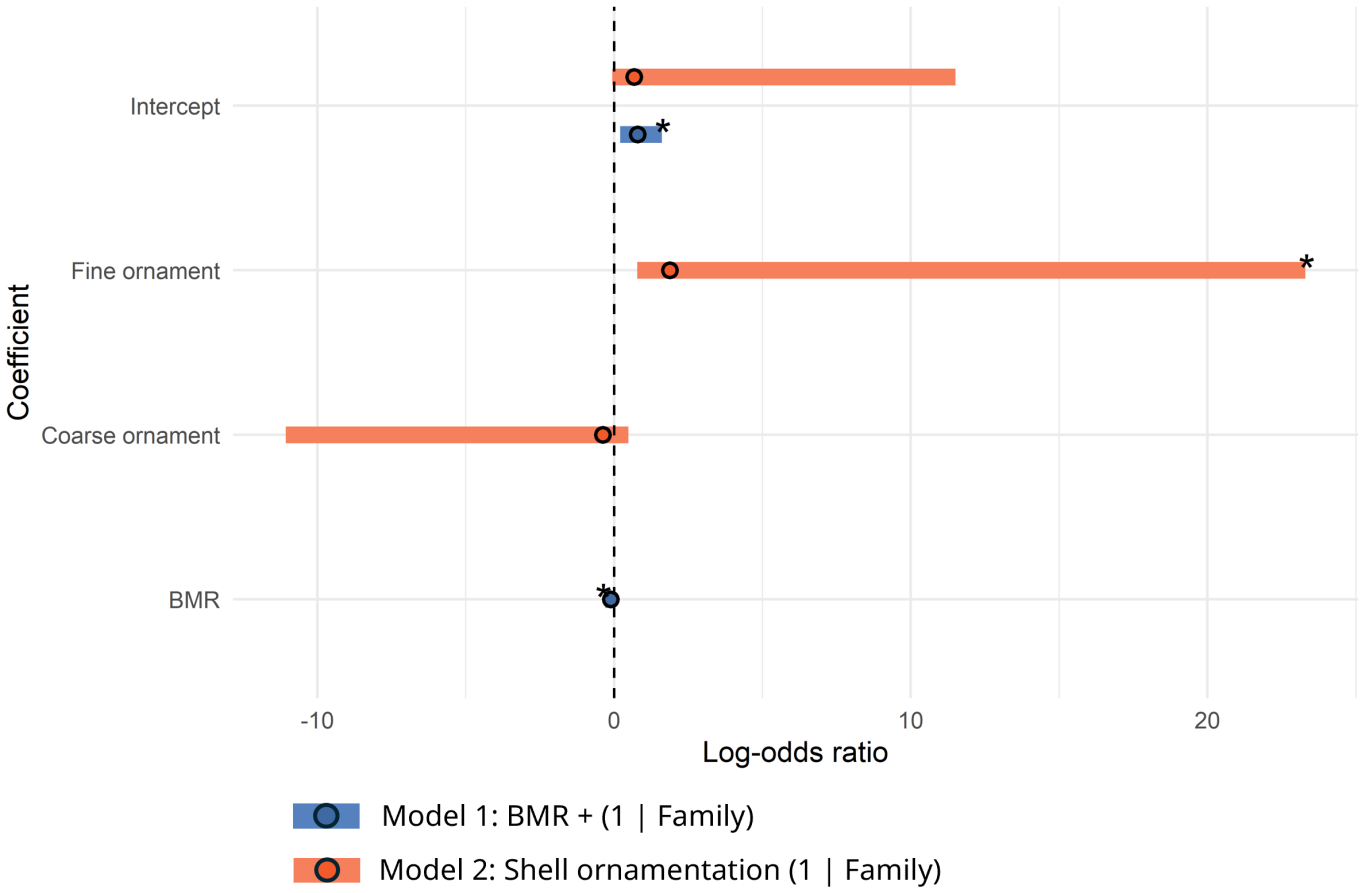
Log-odds ratios forest plot by model (bivalves group-specific traits). The horizontal bars represent 95% confidence intervals. The intercept of the models represents the log-odds of survival when BMR equals 0 and the categorical traits of each model are set to their reference level (if the model considers any). The asterisk highlights effect sizes for which their confidence intervals do not overlap 0. Positive values indicate a proportional relationship with survival, and negative values indicate an inverse relationship with survival.

When analyzing bivalve-specific traits, two models met the selection criteria: one included only BMR, while the other included only shell ornamentation (with ‘smooth ornament’ as the reference category; see Table 1). Model averaging estimated that the odds of survival decreased by approximately 4% per 1 mW increase in BMR (model-averaged log-odds ratio: −0.037, CI [−0.209, −0.010]; see models in Fig. 4; Table 3). Similarly, for shell ornamentation, model-averaged results suggest the odds of survival of fine ornamented species are approximately 3 times higher than smooth ornamented species (model-averaged log-odds ratio: 1.241, CI [0.320–3.444]). However, the uncertainty of this effect size is considerably high as shown by its wide confidence interval. Additionally, when BMR is treated as a categorical predictor, only model 1 is retained, as the ΔAICc score of the second model becomes greater than 2 and violates this selection criterion. For gastropods, no evidence of association between group-specific traits and survival was found (Table S12), therefore no logistic regression models were developed for this dataset.

To evaluate if over splitting of some categorical traits could impact the capacity of our models to detect specific patterns for the class-specific trait datasets, we ran our models on versions of these datasets with reduced number of categories in certain traits. Feeding type in gastropods, and feeding type and ridges morphology in bivalves, were grouped into broader categories (Tables S5–S6). Again, no initial evidence for statistically significant associations in gastropods was found (Table S13). However, feeding type in bivalves initially showed a statistically significant association with survival (ridges morphology did not; Table S14) in this alternative analysis. Then, as above, models were analyzed including feeding type as an additional predictor to the four predictors used originally. No models considering this trait as a predictor passed the selection criteria, and models obtained for these alternative analyses considered the same predictors as the ones obtained in the original analysis, yielding very similar results (Fig. S3).

## Discussion

Among all the traits we considered, BMR was the only trait that showed a consistent association with species survival across both bivalves and gastropods from the late Neogene of the western Atlantic. In particular, we found that for every 1mW increase in BMR there was an average ∼11% decrease in the odds of survival overall. No differences in statistical significance were observed when this trait was treated as a categorical variable. This result was robust to differences in the number of bivalve and gastropod taxa considered and potential multicollinearity among certain predictors, and reiterates the pattern documented by Strotz et al. (2018), who found that higher metabolic rates in both bivalves and gastropods from this period were associated with extinction. This pattern was also found to be consistent when analyzing bivalves in isolation within the shared-traits dataset, and when analyzing this trait in the bivalve-specific traits dataset, although the estimated effect is weaker in this last case, reflecting some uncertainty from models that did not consider this trait. Given none of the models analyzing gastropods in isolation passed our model diagnostics and selection criteria, we are not able to provide concrete conclusions about the effects of BMR in this group specifically. This could be due to our relatively small sample size for this group, which may not provide enough numerical power to evaluate this effect while considering families as a random effect. Increasing the number of taxa per taxonomic family in future research that applies this, and similar methodologies, would likely help overcome this issue and provide clearer signals.

When it comes to estimating BMR values, an important caveat concerns the assumption that basal metabolic rate and body mass scale with a consistent exponent—most notably, the widely debated ‘3/4 power law’ of body mass. This happens to remain an active area of research with no clear consensus (Glazier, 2005; Isaac & Carbone, 2010; White & Kearney, 2013; White & Marshall, 2023). While abundant evidence both supports and challenges this hypothesis, part of the debate centers on how ‘universal’ this exponent truly is. A central tendency around a scaling factor of 3/4 has long been reported (Hemmingsen, 1960; Isaac & Carbone, 2010; Moses et al., 2008; White et al., 2022), yet substantial variability has also been documented at both interspecific (*e.g.,* Glazier, 2005; Glazier, 2010; White & Kearney, 2013) and intraspecific levels (*e.g.,* Glazier, 2005). For bivalves and gastropods, previous studies support a central tendency near 3/4 (Glazier, 2005; Glazier, 2010), though with intraspecific variation, particularly among gastropods (Glazier, 2022). The robustness of our results increases as the true interspecific variance of this exponent decreases. Further, similarly to what Strotz et al. (2018) discussed, if this value is similar among species, a change in the exponent used would not affect these results (see Supplementary Material 1 of Strotz et al., 2018: p.3). However, if metabolic scaling factors differ considerably among populations or species, then these species-level averaged BMR values—regardless of the exponent used—may not fully capture the overall variability of metabolic scaling both within and among species. Furthermore, since BMR values for species were calculated using estimations of shell biomass obtained from maximum linear dimension measurements as a proxy for body size, it is difficult to separate the effects of these factors in this case. If metabolic scaling exponents differ considerably among species, the pattern observed herein would be more closely related to body size than metabolic rate itself, and our results would suggest decreasing odds of survival with increasing body size, contrary to the findings obtained by Monarrez, Heim & Payne (2023). This discrepancy could stem from differences in the taxonomic, spatial, and temporal scales of the two studies. Moreover, body size data in Monarrez, Heim & Payne (2023) were collected at the genus level, contrary to the species-level estimations from Strotz et al. (2018), which may further contribute to differences in the observed patterns and complicate direct comparisons with the results reported here. Nonetheless, if the observed selectivity pattern is truly linked to BMR, while acknowledging that this trait may actually encompass the influence of multiple physiological and ecological traits (Seibel & Drazen, 2007; Strotz et al., 2018), the consistent association between increased BMR and higher odds of extinction found in bivalves, as well as when bivalves are compared to gastropods, supports a link between predictions of the Metabolic Theory of Ecology (MTE, Brown et al., 2004) and species-level processes at macroevolutionary timescales. MTE proposes one of the drivers of changes in species diversity is the relationship between speciation and temperature, and argues this relation is a consequence of processes occurring at the organismal and population levels (Brown et al., 2004). However, incorporating a metabolic mechanism that explains extinction into the theory may allow MTE to more fully link individual-level processes to macroevolutionary patterns at the species level. Overall, our results show that differences of only a few milliwatts in BMR among taxa had considerable implications for the survival of the species considered herein during the late Cenozoic. The increased vulnerability of species with higher energetic requirements may be explained by the gradual decrease in primary productivity and nutrients in the waters of the northwestern Atlantic—particularly the Caribbean Sea—caused by the final closure of the Isthmus of Panama around 3.5-3 Ma (Allmon et al., 1996; Allmon, 2001; Schneider & Schmittner, 2006; Jaramillo, 2018). This hypothesis has also been discussed by Gavirneni, Ivany & Reddin (2025), who found a similar pattern, although not statistically significant, in their data for the Pleistocene. Such a reduction in nutrient availability may have imposed a major, though not exclusive, environmental pressure that contributed to the extinction patterns observed in our data. Other proposed mechanisms, less directly related to the patterns observed herein for this trait, include drops in temperature due to the onset of the Northern Hemisphere glaciation (Allmon, 2001) and sea-level changes linked to the intensified Pleistocene glacial cycles (Berger & Jansen, 1994; Jackson, 1994). However, evidence supporting these hypotheses is weaker, and a productivity-related explanation remains among the most plausible ones (O’Dea et al., 2007).

Another caveat that must be emphasized when interpreting our results is that they are based on a limited range of taxa and only cover a specific period and region. Further, it is important to note that our findings do not suggest metabolism is the sole factor associated with extinction selectivity in this case, as previous studies have demonstrated that a range of factors influence extinction patterns in mollusks, including nutrient supply and primary productivity (*e.g.*, Allmon, 2001; Todd et al., 2002), feeding (*e.g.*, Todd et al., 2002; Mondanaro, Dominici & Danise, 2024), life habit (*e.g.*, Rivadeneira & Marquet, 2007; Aberhan & Kiessling, 2015; Orzechowski et al., 2015), geographic range (*e.g.*, Rivadeneira & Marquet, 2007; Harnik, 2011; Finnegan et al., 2015; Orzechowski et al., 2015; Saupe et al., 2015; Monarrez, Heim & Payne, 2023; Malanoski et al., 2024; Mondanaro, Dominici & Danise, 2024), ecological niches and niche breadth (*e.g.*, Saupe et al., 2014a; Saupe et al., 2014b; Saupe et al., 2015; Mondanaro, Dominici & Danise, 2024), body size (*e.g.*, Rivadeneira & Marquet, 2007; Monarrez, Heim & Payne, 2023) and other physiology-related properties (Malanoski et al., 2024).

When it comes to life habit, our analyses had limited ability to evaluate its effect on survival, and it remains as an unresolved question herein due to the lack of conclusive findings in our results. None of our best-supported models included life habit as a predictor, since candidate models incorporating this trait failed to meet our selection and diagnosis criteria due to numerical instability and convergence issues. No consistent evidence for an effect of this trait was found either when levels with few observations (boring and semi-infaunal) were grouped to avoid perfect-prediction issues. From these results, it is clear that, at least in our dataset, this trait not only failed to improve model fit but hindered it in most cases. These numerical instability issues may stem from the effects of various factors, including small sample sizes of certain levels (boring and semi-infaunal), high collinearity of this trait with other predictors, and overparameterization relative to the random-effects structure. Nonetheless, while some studies have shown that life habit has played a role in mediating taxon survival at different temporal and spatial scales (*e.g.*, Rivadeneira & Marquet, 2007; Leonard-Pingel, Jackson & O’Dea, 2012; Orzechowski et al., 2015), certain works focusing on similar contexts within the late Cenozoic have found variable results (*e.g.*, Todd et al., 2002; Leonard-Pingel, Jackson & O’Dea, 2012).

Although one of our best-supported models for the shared traits dataset considers an interaction term between BMR and shell composition, this effect is not statistically significant. A similar result is found when bivalves are analyzed in isolation within the same dataset. In that case, an additional model includes a main-effect term for shell composition, but the associated effect size is more uncertain than those of other predictors and is also not statistically significant. Overall, these results do not support the hypothesis that shell composition may have influenced extinction selectivity in these species during the late Cenozoic. It is commonly expected that aragonitic organisms are more vulnerable to climate-associated pressures, such as ocean acidification, due to the higher solubility of aragonite relative to low-Mg calcite (Andersson, Mackenzie & Bates, 2008; Ries, 2011), and this vulnerability may be linked to extinction patterns at macroevolutionary timescales (Kiessling, Aberhan & Villier, 2008). However, since no major ocean acidification events have been identified during this period (Orzechowski et al., 2015), based on analyses by Hannisdal & Peters (2011), our results are consistent with the expectation of no selectivity relative to such environmental pressure during this time interval. By contrast, other properties including crystal structure, shell thickness and the proportion of organic matrix in the shell may have played a more important role in this case (Harper, 2000), given that these factors associated with shell strength (Goswami, 2021; Cartwright, Checa & Vendrasco, 2024), have been important predation-related adaptations throughout the evolutionary history of molluscan biomineralization (Cartwright, Checa & Vendrasco, 2024). No models considering this trait were retained when oysters were treated as exclusively calcitic and three levels of shell composition were evaluated.

Shell ornamentation was the only bivalve-specific trait among the three tested in mixed-effects models that showed some evidence of a statistically significant association with survival and extinction patterns in our data. However, the uncertainty in the estimated effect size is substantial. The weak signal observed here might still represent an indication of an influence of different degrees of shell ornamentation on survival, which could be associated with potential differences in burrowing efficiency or shell thickness and their responses to multiple selective pressures, including predation (Hansen et al., 1999; Cartwright, Checa & Vendrasco, 2024) and environmental perturbations (Stanley, 1988; Sälgeback, 2006). Nevertheless, evaluating these relationships will require further investigation encompassing a wider range of taxa. While the other two traits—organism/substrate relationship and mobility—initially showed associations with survival in chi-squared tests of independence, all models incorporating these traits exhibited numerical instability or convergence issues, preventing a full evaluation of these relationships at this time. While these results do not rule out a potential role of these traits in survival, further data is needed to assess their influence more confidently in this system.

Other ecological traits linked to predation and predation defense such as feeding type and various morphological structures of the shell (*e.g.*, varices and siphonal canal), show little relation with extinction selectivity in the taxa examined. This could suggest some of these traits, sometimes posited to represent important organismic adaptations, may not have played a prominent role in long term species survival, particularly during the profound environmental changes of the last ∼3 Ma in this region (*e.g.*, the Northern Hemisphere glaciation: Allmon, 2001; lengthening of glacial cycles during the Mid-Pleistocene: Legrain, Parrenin & Capron, 2023; sea-level changes linked to these intensified Pleistocene glacial cycles: Berger & Jansen, 1994; Jackson, 1994). This could be because anti-predatory traits might be less important during times of substantial climate change. Further, this implies that processes demonstrated to impact the survival of organisms may not always scale up to explain the differential survival of species (see Jablonski, 2008; Strotz et al., 2018 and references therein).

Our data represents less than 3% of the known species of the western Atlantic, and despite the robustness of our analyses, this inevitably limits the generalizability of our results to some extent and introduces potential sampling biases. We addressed biases arising from the unbalanced representation of bivalves and gastropods, as well as from the hierarchical taxonomic structure of our data (class and family) and its associated variance. Although our results appear robust to some key sources of bias and our sensitivity tests suggest that these factors likely did not distort the observed patterns, other sources of bias may remain unaccounted for. These additional biases could have particularly influenced the patterns observed for BMR and shell composition. For instance, the fact that we considered only a few predominantly calcitic taxa (four oyster species) relative to the other groups could have affected our ability to detect a clearer selectivity signal for shell composition and therefore the pattern of no selectivity for this trait should be interpreted with caution. Furthermore, although we provide a quantitative estimate of the decrease in survival odds associated with increases in BMR, these estimates should be refined using a wider range of taxa from the late Cenozoic, as they are based on a limited number of species and may therefore be biased in magnitude. While we cannot rule out sample-size effects entirely, it is more likely that larger datasets would reveal additional patterns rather than overturn the patterns observed here. Overall, our findings contribute to the broader understanding of traits that serve as important predictors of extinction risk in Pliocene to recent bivalves and gastropods from the western Atlantic. Moreover, the methodology applied in this study is applicable to future trait-based research, and the approach described herein provides a framework for applying these methods to mixed types of trait data, making them more flexible and broadly applicable.

## Conclusions

Our results provide evidence that certain traits may be associated with the observed patterns of extinction selectivity in the late Cenozoic species considered herein, while others are not. Particularly, our analyses support the hypothesis that BMR is associated with the extinction of species, corroborating the findings from Strotz et al. (2018), and quantifying an average decrease of ∼11% in the odds of survival of species for every 1 mW-increase in BMR. Conversely, the hypothesized relation with survival/extinction of other traits such as the presence of varices in gastropods, shell fixation, and shell composition, was not supported by our results. These may represent cases of organismal traits that did not influence overall species survival/extinction during the studied interval. It is likely the interaction between intrinsic biological mechanisms and the abiotic environment may determine the extent to which certain processes at lower hierarchical levels (intraspecific) influence patterns at higher hierarchical (interspecific) levels (Jablonski, 2008). Therefore, under different environmental conditions, or when different taxa are considered, one might posit different patterns would prevail. Thus, understanding the relationships between specific organismal-level traits, and how these traits respond to environmental changes, is key for obtaining insights into the mechanisms underlying extinction processes.

##  Supplemental Information

10.7717/peerj.20715/supp-1 Supplemental Information 1 Subset analysis. Balanced data replicates–Shared traits dataset–BMR main effect Model 1 Estimation of effect size uncertainty via subsampling - 100 balanced replicates (38 bivalves - 38 gastropods). The red arrow indicates the true log-odds ratio, the yellow arrow indicates the mean log-odds ratio of the distribution of resampled estimates, and blue dotted lines indicate the 95% confidence interval of this distribution.

10.7717/peerj.20715/supp-2 Supplemental Information 2 Subset analysis. Balanced data replicates –Shared traits dataset –BMR main effect Model 2 Estimation of effect size uncertainty via subsampling - 100 balanced replicates (38 bivalves - 38 gastropods). The red arrow indicates the true log-odds ratio, the yellow arrow indicates the mean log-odds ratio of the distribution of resampled estimates, and blue dotted lines indicate the 95% confidence interval of this distribution.

10.7717/peerj.20715/supp-3 Supplemental Information 3 Shared-traits dataset BMR: Basal metabolic rate. BMR data obtained from Strotz et al. (2018) and is measured in watts (W). Life habit data obtained from the Paleobiology Database (PBDB) and associated references for this data are provided. Shell composition data for oysters (family: Ostreidae) obtained from Checa, Harper & González-Segura (2018), Hautmann (2006) and Stenzel (1964). Shell composition data for the rest of taxa obtained from PBDB.

10.7717/peerj.20715/supp-4 Supplemental Information 4 Bivalve-specific traits dataset BMR: Basal metabolic rate. Organism/Substrate Relationship - ER: Epifaunal recliner, EP: Epifaunal, SI: Semi-infaunal, IS: Infaunal siphonate, IA: Infaunal asiphonate, WN: Nestler on or within hard substrates, WB: Borer, nestling in hard substrate. Mobility - IM: Immobile, SE: Sedentary, MA: Actively mobile, and SW: Swimming. Feeding type - SU: Suspension feeder, DS: Surface deposit feeder, and DC: Chemosymbiotic deposit feeder. Shell fixation - UN: Unattached, BA: Bysally attached, and CE: Cemented. BMR data obtained from Strotz et al. (2018) and is measured in watts (W).

10.7717/peerj.20715/supp-5 Supplemental Information 5 Gastropod-specific traits BMR: Basal metabolic rate. Feeding type - CP: predatory carnivores, CB: browsing canivores, HM: herbivores on fine-grained substrates, HR: herbivores on rock, rubble or coral substrates, microalgivores, HP: herbivores on plant or algal substrates, micro-and macroalgivores and detritivores on macroalgal and seagrass substrates, SU: Suspension feeders. Missing vaues are labeled as ’undetermined’. BMR data obtained from Strotz et al. (2018) and is measured in watts (W).

10.7717/peerj.20715/supp-6 Supplemental Information 6 Shared-traits dataset - alternative BMR: Basal metabolic rate. BMR data obtained from Strotz et al. (2018) and is measured in watts (W). Life habit and shell composition data obtained from the Paleobiology Database (PBDB) and associated references for this data are provided.

10.7717/peerj.20715/supp-7 Supplemental Information 7 Bivalves-specific traits - alternative grouping Feeding type and ridges morphology are re-classified in this version. Surface deposit feeders (labeled as ”DS” in **Table S2**) and chemosymbiotic deposit feeders (labeled originally as “DC”) were grouped into one category of deposit feeders, labeled as ”DF” in this version. Ridges originally classified as “quasi-commarginal” in **Table S2** were included in the “commarginal” category, and “quasi-radial” ridges were considered as “radial”. BMR: Basal metabolic rate; BMR data obtained from Strotz et al. (2018) and is measured in watts (W). Organism/Substrate Relationship, Mobility, Shell fixation and Feeding type data were obtained from the Neogene Marine Biota of Tropical America (NMITA) database (https://nmita.rsmas.miami.edu/nmita.htm). Detailed descriptions of each trait and its categories can be found here: https://nmita.rsmas.miami.edu/database/mollusc/mollusclifestyles.htm. Ridges morphology and shell ornamentation data were obtained from examination of specimens at the Paleontological Research Institution (PRI) and Florida Museum of Natural History (FLMNH) collections.

10.7717/peerj.20715/supp-8 Supplemental Information 8 Gastropod-specific traits (alternative version) Feeding type is re-classified in this version. Predatory and browsing carnivores (CP and CB, respectively in **Table S3** ) are clumped into a single category of carnivores (C) in this table. Herbivores on fine-grained substrates (HM) and herbivores on plant or algal substrates (HP) were grouped into a single category of herbivores (H). Species with more than one feeding type were not included in these groups. Missing vaues are labeled as ’undetermined’. BMR: Basal metabolic rate; BMR data obtained from Strotz et al. (2018) and is measured in watts (W). Organism/Substrate Relationship, Mobility, Shell fixation and Feeding type data were obtained from the Neogene Marine Biota of Tropical America (NMITA) database (https://nmita.rsmas.miami.edu/nmita.htm). Detailed descriptions of each trait and its categories can be found here: https://nmita.rsmas.miami.edu/database/mollusc/mollusclifestyles.htm. Ridges morphology and shell ornamentation data were obtained from examination of specimens at the Paleontological Research Institution (PRI) and Florida Museum of Natural History (FLMNH) collections.

10.7717/peerj.20715/supp-9 Supplemental Information 9 List of all models considered BMR stands for basal metabolic rate. Chi-squared statistics and p-values for each trait were obtained from type III ANOVA tests performed on logistic regression models. Corrected Akaike Information Criterion scores (AICc) for each model are listed. Log-odds ratios are provided for each trait and for the intercept of each model. Intercepts represent the log-odds of survival when BMR is equal to 0 and/or the categorical traits of a model are set to their reference level. Interaction terms are indicated with an ‘x’, the shell composition reference level used was: ‘aragonite’, and the shell ornamentation reference level used was: ‘smooth ornament’. Levels of statistical significance are labeled as follows: * *α* < 0.05 and ** *α* < 0.01. Models that passed our selection criteria are highlighted in bold.

10.7717/peerj.20715/supp-10 Supplemental Information 10 List of all models evaluated - BMR treated as a categorical variable BMR stands for basal metabolic rate. Chi-squared statistics and p-values for each trait were obtained from type III ANOVA tests performed on logistic regression models. Corrected Akaike Information Criterion scores (AICc) for each model are listed. Log-odds ratios are provided for each trait and for the intercept of each model. Intercepts represent the log-odds of survival when BMR is equal to 0 and/or the categorical traits of a model are set to their reference level. Interaction terms are indicated with an ‘x’, the reference level used for BMR was: ‘low’, the shell composition reference level used was: ‘aragonite’, and the reference level used for shell ornamentation was: ‘smooth ornament’. Levels of statistical significance are labeled as follows: * *α* < 0.05 and ** *α* < 0.01. Models that passed our selection criteria are highlighted in bold.

10.7717/peerj.20715/supp-11 Supplemental Information 11 List of all models evaluated –evaluating three levels of shell composition BMR stands for basal metabolic rate. Chi-squared statistics and p-values for each trait were obtained from type III ANOVA tests performed on logistic regression models. Corrected Akaike Information Criterion scores (AICc) for each model are listed. Log-odds ratios are provided for each trait and for the intercept of each model. Intercepts represent the log-odds of survival when BMR is equal to 0. Levels of statistical significance are labeled as follows: * *α* ¡ 0.05 and ** *α* ¡ 0.01. Models that passed our selection criteria are highlighted in bold.

10.7717/peerj.20715/supp-12 Supplemental Information 12 List of all models considered –boring and semi-infaunal taxa grouped BMR stands for basal metabolic rate. Chi-squared statistics and p-values for each trait were obtained from type III ANOVA tests performed on logistic regression models. Corrected Akaike Information Criterion scores (AICc) for each model are listed. Log-odds ratios are provided for each trait and for the intercept of each model. Intercepts represent the log-odds of survival when BMR is equal to 0 and/or the categorical traits of a model are set to their reference level. Interaction terms are indicated with an ‘x’, the shell composition reference level used was: ‘aragonite’. Levels of statistical significance are labeled as follows: * *α* ¡ 0.05 and ** *α* ¡ 0.01. Models that passed our selection criteria are highlighted in bold.

10.7717/peerj.20715/supp-13 Supplemental Information 13 Chi-squared tests of independence for bivalve-specific traits Statistically significant associations of traits with survival are highlighted in bold.

10.7717/peerj.20715/supp-14 Supplemental Information 14 Chi-squared tests of independence for gastropod-specific traits

10.7717/peerj.20715/supp-15 Supplemental Information 15 Chi-squared tests of independence for alternative dataset of gastropod-specific traits In this alternative dataset, predatory and browsing carnivores (CP and CB respectively in Table S3) were clumped into a single category of carnivores (C). Similarly, herbivores on fine-grained substrates (HM) and herbivores on plant or algal substrates (HP) were grouped into a single category of herbivores (H).

10.7717/peerj.20715/supp-16 Supplemental Information 16 Chi-squared tests of independence for alternative dataset of bivalve-specific traits In this alternative dataset, ridges morphologies and feeding types were reclassified. Ridges classified as “quasi-commarginal” in bivalves originally, were included in the “commarginal” category, and “quasi-radial” ridges were considered as “radial”. Regarding feeding type in this group, surface deposit feeders (labeled as DS in Table S2) and chemosymbiotic deposit feeders (DC in Table S2) were grouped into one category of deposit feeders (DF). Statistically significant associations of traits with survival are highlighted in bold.

## Additional Information and Declarations

### Data Availability

The following information was supplied regarding data availability: The data is available at at Dryad: Rojas Ariza, Daniel Eduardo; Strotz, Luke C.; Lieberman, Bruce S. (2026). Differences in extinction selectivity and their relationship to functional traits in late Cenozoic molluscs [Dataset]. Dryad. https://doi.org/10.5061/dryad.k6djh9wgq.

## References

[ref-1] Abbott RT, Dance SP (1986). Compendium of seashells: a color guide to more than 4,200 of the world’s marine shells.

[ref-2] Aberhan M, Alroy J, Fürsich FT, Kiessling W, Kosnik M, Madin J, Patzkowsky M, Wagner PJ (2004). Ecological attributes of marine invertebrates. Paleobiology Database reference #9941. https://paleobiodb.org/classic/app/refs#display=9941.

[ref-3] Aberhan M, Kiessling W (2015). Persistent ecological shifts in marine molluscan assemblages across the end-Cretaceous mass extinction. Proceedings of the National Academy of Sciences of the United States of America.

[ref-4] Albano PG, Schultz L, Wessely J, Taviani M, Dullinger S, Danise S (2024). The dawn of the tropical Atlantic invasion into the Mediterranean Sea. Proceedings of the National Academy of Sciences of the United States of America.

[ref-5] Allison P (2008). Convergence failures in logistic regression. SAS Global Forum.

[ref-6] Allmon WD (1989). Paleontological completeness of the record of lower tertiary mollusks, US Gulf and Atlantic coastal plains: implications for phylogenetic studies. Historical Biology.

[ref-7] Allmon WD (2001). Nutrients, temperature, disturbance, and evolution: a model for the late Cenozoic marine record of the western Atlantic. Palaeogeography, Palaeoclimatology, Palaeoecology.

[ref-8] Allmon WD, Emslie SD, Jones DS, Morgan GS (1996). Late Neogene oceanographic change along Florida’s west coast: evidence and mechanisms. Journal of Geology.

[ref-9] Allmon WD, Rosenberg G, Portell RW, Schindler KS (1993). Diversity of Atlantic Coastal Plain mollusks since the Pliocene. Science.

[ref-10] Andersson AJ, Mackenzie FT, Bates NR (2008). Life on the margin: implications of ocean acidification on Mg-calcite, high latitude and cold-water marine calcifiers. Marine Ecology Progress Series.

[ref-11] Arnold TW (2010). Uninformative parameters and model selection using Akaike’s Information Criterion. Journal of Wildlife Management.

[ref-12] Balthasar U, Cusack M (2015). Aragonite–calcite seas: quantifying the gray area. Geology.

[ref-13] Bartoń K (2024). https://CRAN.R-project.org/package=MuMIn.

[ref-14] Bates D, Mächler M, Bolker B, Walker S (2015). Fitting linear mixed-effects models using *lme4*. Journal of Statistical Software.

[ref-15] Benjamini Y, Hochberg Y (1995). Controlling the false discovery rate: a practical and powerful approach to multiple testing. Journal of the Royal Statistical Society B.

[ref-16] Berger WH, Jansen E (1994). Mid-Pleistocene climate shift—the Nansen connection. Geophysical Monograph Series.

[ref-17] Brown JH, Gillooly JF, Allen AP, Savage VM, West GB (2004). Toward a metabolic theory of ecology. Ecology.

[ref-18] Budd AF, Foster CT, Dawson JP, Johnson KG (2001). The Neogene marine biota of tropical America (NMITA) database: accounting for biodiversity in paleontology. Journal of Paleontology.

[ref-19] Burke KD, Williams JW, Chandler MA, Haywood AM, Lunt DJ, Otto-Bliesner BL (2018). Pliocene and Eocene provide best analogs for near-future climates. Proceedings of the National Academy of Sciences of the United States of America.

[ref-20] Burnham KP, Anderson DR (2002). Model selection and multimodel inference: a practical information-theoretic approach.

[ref-21] Carter JG, Barrera E, Tevesz MJS (1998). Thermal potentiation and mineralogical evolution in the Bivalvia (Mollusca). Journal of Paleontology.

[ref-22] Cartwright JH, Checa AG, Vendrasco MJ (2024). Arms and the mollusc: an evolutionary arms race has produced armor based on molluscan biomineralization. MRS Bulletin.

[ref-23] Checa AG, Harper EM, González-Segura A (2018). Structure and crystallography of foliated and chalk shell microstructures of the oyster *Magallana*: the same materials grown under different conditions. Scientific Reports.

[ref-24] Chen SY, Feng Z, Yi X (2017). A general introduction to adjustment for multiple comparisons. Journal of Thoracic Disease.

[ref-25] Chichorro F, Urbano Tenorio F, Teixeira D, Väre H, Pinto T, Brummitt N, He X, Hochkirch A, Hyvönen J, Kaila L, Juslén A, Cardoso P (2022). Trait-based prediction of extinction risk across terrestrial taxa. Biological Conservation.

[ref-26] Collins KS, Edie SM, Hunt G, Roy K, Jablonski D (2018). Extinction risk in extant marine species integrating palaeontological and biodistributional data. Proceedings of the Royal Society B: Biological Sciences.

[ref-27] Dowsett HJ, Robinson MM, Foley KM, Herbert TD (2021). The Yorktown formation: improved stratigraphy, chronology, and paleoclimate interpretations from the U.S. Mid-Atlantic Coastal Plain. Geosciences.

[ref-28] Dudoit S, Van der Laan MJ (2008). Multiple testing procedures with applications to genomics.

[ref-29] Eklöf J, Austin Å, Bergström U, Donadi S, Eriksson BD, Hansen J, Sundblad G (2017). Size matters: relationships between body size and body mass of common coastal, aquatic invertebrates in the Baltic Sea. PeerJ.

[ref-30] Finnegan S, Anderson SC, Harnik PG, Simpson C, Tittensor DP, Byrnes JE, Finkel ZV, Lindberg DR, Liow LH, Lockwood R, Lotze HK, McClain CR, McGuire JL, O’Dea A, Pandolfi JM (2015). Paleontological baselines for evaluating extinction risk in the modern oceans. Science.

[ref-31] Gavirneni S, Ivany LC, Reddin CJ (2025). Burning calories, burning ocean: metabolic rate in bivalves as a predictor of extinction selectivity through time and during rapid global warming. Paleobiology.

[ref-32] Gillooly JF, Brown JH, West GB, Savage VM, Charnov EL (2001). Effects of size and temperature on metabolic rate. Science.

[ref-33] Glazier DS (2005). Beyond the ‘3/4-power law’: variation in the intra-and interspecific scaling of metabolic rate in animals. Biological Reviews.

[ref-34] Glazier DS (2010). A unifying explanation for diverse metabolic scaling in animals and plants. Biological Reviews.

[ref-35] Glazier DS (2022). Variable metabolic scaling breaks the law: from ‘Newtonian’ to ‘Darwinian’ approaches. Proceedings of the Royal Society B: Biological Sciences.

[ref-36] Goswami A (2021). A comparative study on the mechanical and structural design of nacre in gastropod and bivalve molluscs. Journal of the Mechanical Behavior of Biomedical Materials.

[ref-37] Hannisdal B, Peters SE (2011). Phanerozoic earth system evolution and marine biodiversity. Science.

[ref-38] Hansen TA, Kelley PH, Melland VD, Graham SE (1999). Effect of climate-related mass extinctions on escalation in molluscs. Geology.

[ref-39] Harnik PG (2011). Direct and indirect effects of biological factors on extinction risk in fossil bivalves. Proceedings of the National Academy of Sciences of the United States of America.

[ref-40] Harnik PG, Lotze HK, Anderson SC, Finkel ZV, Finnegan S, Lindberg DR, Liow LH, Lockwood R, McClain CR, McGuire JL, O’Dea A, Pandolfi JM, Simpson C, Tittensor DP (2012). Extinctions in ancient and modern seas. Trends in Ecology & Evolution.

[ref-41] Harper EM (2000). Are calcitic layers an effective adaptation against shell dissolution in the Bivalvia?. Journal of Zoology.

[ref-42] Harper EM, Palmer TJ, Alphey JR (1997). Evolutionary response by bivalves to changing Phanerozoic sea-water chemistry. Geological Magazine.

[ref-43] Hartig F (2024). https://cran.r-project.org/package=DHARMa.

[ref-44] Hautmann M (2006). Shell mineralogical trends in epifaunal Mesozoic bivalves and their relationship to seawater chemistry and atmospheric carbon dioxide concentration. Facies.

[ref-45] Hemmingsen AM (1960). Energy metabolism as related to body size and respiratory surface, and its evolution. Report of Steno Memorial Hospital.

[ref-46] Hendricks H, Stigall A, Lieberman B (2015). The digital atlas of ancient life: delivering information on paleontology and biogeography *via* the web. Palaeontologia Electronica.

[ref-47] Hendy A, Aberhan M, Alroy J, Clapham M, Kiessling W, Lin A, LaFlamme M (2009). Unpublished ecological data in support of GSA 2009 abstract: a 600 million year record of ecological diversification. Paleobiology Database reference #29272. https://paleobiodb.org/classic/app/refs#display=9941.

[ref-48] Huang S, Edie SM, Collins KS, Crouch NM, Roy K, Jablonski D (2023). Diversity, distribution and intrinsic extinction vulnerability of exploited marine bivalves. Nature Communications.

[ref-49] Isaac NJ, Carbone C (2010). Why are metabolic scaling exponents so controversial? Quantifying variance and testing hypotheses. Ecology Letters.

[ref-50] Jablonski D (2005). Mass extinctions and macroevolution. Paleobiology.

[ref-51] Jablonski D (2008). Species selection: theory and data. Annual Review of Ecology, Evolution, and Systematics.

[ref-52] Jablonski D, Hunt G (2006). Larval ecology, geographic range, and species survivorship in Cretaceous mollusks: organismic *versus* species-level explanations. The American Naturalist.

[ref-53] Jablonski D, Raup DM (1995). Selectivity of end-Cretaceous marine bivalve extinctions. Science.

[ref-54] Jackson JBC (1994). Community unity?. Science.

[ref-55] Jaramillo C, Hoorn C, Perrigo A, Antonelli A (2018). Evolution of the Isthmus of Panama: biological, paleoceanographic and paleoclimatological implications. Mountains, climate and biodiversity.

[ref-56] Khan BM, Liu Y (2019). Marine mollusks: food with benefits. Comprehensive Reviews in Food Science and Food Safety.

[ref-57] Kidwell SM, Flessa KW (1995). The quality of the fossil record: populations, species, and communities. Annual Review of Ecology and Systematics.

[ref-58] Kiessling W (2004). Ecology opinions. Paleobiology Database. Reference #9940. https://paleobiodb.org/classic/app/refs#display=9940.

[ref-59] Kiessling W, Aberhan M, Villier L (2008). Phanerozoic trends in skeletal mineralogy driven by mass extinctions. Nature Geoscience.

[ref-60] Kiessling W, Reddin CJ, Dowding EM, Dimitrijević D, Raja NB, Kocsis ÁT (2025). Marine biological responses to abrupt climate change in deep time. Paleobiology.

[ref-61] Klompmaker AA, Kelley PH (2015). Shell ornamentation as a likely exaptation: evidence from predatory drilling on Cenozoic bivalves. Paleobiology.

[ref-62] Knoll AH, Bambach RK, Canfield DE, Grotzinger JP (1996). Comparative earth history and Late Permian mass extinction. Science.

[ref-63] Legrain E, Parrenin F, Capron E (2023). A gradual change is more likely to have caused the Mid-Pleistocene Transition than an abrupt event. Communications Earth & Environment.

[ref-64] Leonard-Pingel JS, Jackson JBC, O’Dea A (2012). Changes in bivalve functional and assemblage ecology in response to environmental change in the Caribbean Neogene. Paleobiology.

[ref-65] Lieberman BS, Miller III W, Eldredge N (2007). Paleontological patterns, macroecological dynamics and the evolutionary process. Evolutionary Biology.

[ref-66] Lindberg D, Ponder WF (2001). The influence of classification on the evolutionary interpretation of structure: a re-evaluation of the evolution of the pallial cavity of gastropod molluscs. Organisms Diversity & Evolution.

[ref-67] Lockwood R (2008). Beyond the big five: extinctions as experiments in the history of life. The Paleontological Society Papers.

[ref-68] Lourens LJ, Becker J, Bintanja R, Hilgen FJ, Tuenter E, Van de Wal RSW, Ziegler M (2010). Linear and non-linear response of late Neogene glacial cycles to obliquity forcing and implications for the Milankovitch theory. Climate of the Last Million Years: New Insights from EPICA and Other Records.

[ref-69] Lourens LJ, Hilgen FJ (1997). Long-periodic variations in the earth’s obliquity and their relation to third-order eustatic cycles and late Neogene glaciations. Quaternary International.

[ref-70] Lüdecke D, Ben-Shachar MS, Patil I, Waggoner P, Makowski D (2021). Performance: an R package for assessment, comparison and testing of statistical models. Journal of Open Source Software.

[ref-71] MacKenzie Jr CL, Burrell Jr VG, Rosenfield A, Hobart WL (1997). The history, present condition, and future of the molluscan fisheries of North and Central America and Europe: Vol. 3, Europe.

[ref-72] Malanoski CM, Farnsworth A, Lunt DJ, Valdes PJ, Saupe EE (2024). Climate change is an important predictor of extinction risk on macroevolutionary timescales. Science.

[ref-73] Mazerolle MJ (2023). https://cran.r-project.org/package=AICcmodavg.

[ref-74] McDonald JH (2014). Handbook of biological statistics.

[ref-75] McKinney ML (1997). Extinction vulnerability and selectivity: combining ecological and paleontological views. Annual Review of Ecology, Evolution, and Systematics.

[ref-76] McKinney ML, Briggs DEG, Crowther PR (2001). Selectivity during extinctions. Palaeobiology II.

[ref-77] McRoberts CA, Newton CR (1995). Selective extinction among end-Triassic European bivalves. Geology.

[ref-78] Meinhardt H, Klingler M (1987). A model for pattern formation on the shells of molluscs. Journal of Theoretical Biology.

[ref-79] Mikkelsen PM, Bieler R (2007). Seashells of Southern Florida: living Marine Mollusks of the Florida keys and adjacent regions: bivalves.

[ref-80] Mlambo MC (2014). Not all traits are ‘functional’: insights from taxonomy and biodiversity—ecosystem functioning research. Biodiversity and Conservation.

[ref-81] Monarrez PM, Heim NA, Payne JL (2021). Mass extinctions alter extinction and origination dynamics with respect to body size. Proceedings of the Royal Society B.

[ref-82] Monarrez PM, Heim NA, Payne JL (2023). Reduced strength and increased variability of extinction selectivity during mass extinctions. Royal Society Open Science.

[ref-83] Mondanaro A, Dominici S, Danise S (2024). Response of Mediterranean Sea bivalves to Pliocene–Pleistocene environmental changes. Palaeontology.

[ref-84] Moses ME, Hou C, Woodruff WH, West GB, Nekola JC, Zuo W, Brown JH (2008). Revisiting a model of ontogenetic growth: estimating model parameters from theory and data. The American Naturalist.

[ref-85] Nick TG, Campbell KM, Ambrosius WT (2007). Logistic regression. Topics in biostatistics.

[ref-86] O’Dea A, Jackson JBC, Fortunato H, Smith JT, D’Croz L, Johnson KG, Todd JA (2007). Environmental change preceded Caribbean extinction by 2 million years. Proceedings of the National Academy of Sciences of the USA.

[ref-87] Orzechowski EA, Lockwood R, Byrnes JEK, Anderson SC, Finnegan S, Finkel ZV, Harnik PG, Lindberg DR, Liow LH, Lotze HK, McClain CR, McGuire JL, O’Dea A, Pandolfi JM, Simpson C, Tittensor DP (2015). Marine extinction risk shaped by trait–environment interactions over 500 million years. Global Change Biology.

[ref-88] Payne JL, Heim NA, Knope ML, McClain CR (2014). Metabolic dominance of bivalves predates brachiopod diversity decline by more than 150 million years. Proceedings of the Royal Society B: Biological Sciences.

[ref-89] Payne JL, Truebe S, Nützel A, Chang ET (2011). Local and global abundance associated with extinction risk in late Paleozoic and early Mesozoic gastropods. Paleobiology.

[ref-90] Pereira HM, Leadley PW, Proença V, Alkemade JRM, Scharlemann JPW, Fernandez-Manjarrés JF, Araújo MB, Balvanera P, Biggs R, Cheung WWL, Chini L, Cooper HD, Gilman EL, Guénette S, Hurtt GC, Huntington HP, Mace GM, Oberdorff T, Revenga C, Rodrigues P, Scholes RJ, Sumaila UR, Walpole M (2010). Scenarios for global biodiversity in the 21st century. Science.

[ref-91] Pimiento C, Bacon CD, Silvestro D, Hendy A, Jaramillo C, Zizka A, Meyer X, Antonelli A (2020). Selective extinction against redundant species buffers functional diversity. Proceedings of the Royal Society B: Biological Sciences.

[ref-92] Pinheiro JC, Bates DM (1995). Approximations to the log-likelihood function in the nonlinear mixed-effects model. Journal of Computational and Graphical Statistics.

[ref-93] Porter SM (2010). Calcite and aragonite seas and the *de novo* acquisition of carbonate skeletons. Geobiology.

[ref-94] R Core Team (2024). https://www.R-project.org/.

[ref-95] Reddin CJ, Nätscher PS, Kocsis ÁT, Pörtner HO, Kiessling W (2020). Marine clade sensitivities to climate change conform across timescales. Nature Climate Change.

[ref-96] Rhodes MC, Thayer CW (1991). Mass extinctions: ecological selectivity and primary production. Geology.

[ref-97] Ries JB (2011). Skeletal mineralogy in a high-CO_2_ world. Journal of Experimental Marine Biology and Ecology.

[ref-98] Rivadeneira MM, Marquet PA (2007). Selective extinction of late Neogene bivalves on the temperate Pacific coast of South America. Paleobiology.

[ref-99] Rosenberg G (2024). https://portal.idigbio.org/portal/recordsets/774a153b-e556-47f6-95d1-bab49e61cc58.

[ref-100] Rudman WB (1971). Structure and functioning of the gut in the Bullomorpha (Opisthobranchia). Part 1. Herbivores. Journal of Natural History.

[ref-101] Sälgeback J (2006). Functional morphology of gastropods and bivalves. Doctoral dissertation.

[ref-102] Sánchez Roig M (1926). Contribución a la Paleontología Cubana: Los Equinodermos Fósiles de Cuba. Boletín de Minas.

[ref-103] Saupe EE, Hendricks JR, Portell RW, Dowsett HJ, Haywood A, Hunter SJ, Lieberman BS (2014a). Macroevolutionary consequences of profound climate change on niche evolution in marine molluscs over the past three million years. Proceedings of the Royal Society B: Biological Sciences.

[ref-104] Saupe EE, Hendricks JR, Portell RW, Dowsett HJ, Haywood A, Hunter SJ, Lieberman BS (2014b). https://datadryad.org/dataset/.

[ref-105] Saupe EE, Qiao H, Hendricks JR, Portell RW, Hunter SJ, Soberón J, Lieberman BS (2015). Niche breadth and geographic range size as determinants of species survival on geological time scales. Global Ecology and Biogeography.

[ref-106] Schielzeth H, Dingemanse NJ, Nakagawa S, Westneat DF, Allegue H, Teplitsky C, Réale D, Dochtermann NA, Garamszegi LZ, Araya-Ajoy YG (2020). Robustness of linear mixed-effects models to violations of distributional assumptions. Methods in Ecology and Evolution.

[ref-107] Schneider B, Schmittner A (2006). Simulating the impact of the Panamanian seaway closure on ocean circulation, marine productivity and nutrient cycling. Earth and Planetary Science Letters.

[ref-108] Seibel BA, Drazen JC (2007). The rate of metabolism in marine animals: environmental constraints, ecological demands and energetic opportunities. Philosophical Transactions of the Royal Society B: Biological Sciences.

[ref-109] Seilacher A (1973). Fabricational noise in adaptive morphology. Systematic Zoology.

[ref-110] Seilacher A (1985). Bivalve morphology and function. Studies in Geology.

[ref-111] Singmann H, Bolker B, Westfall J, Aust F (2024). https://cran.r-project.org/package=afex.

[ref-112] Smaal AC, Ferreira JG, Grant J, Petersen JK, Strand Ø (2019). Goods and services of marine bivalves.

[ref-113] Smith JT, Roy K (2006). Selectivity during background extinction: Plio-Pleistocene scallops in California. Paleobiology.

[ref-114] Sperandei S (2014). Understanding logistic regression analysis. Biochemia Medica.

[ref-115] Stanley SM (1969). Bivalve mollusk burrowing aided by discordant shell ornamentation. Science.

[ref-116] Stanley SM (1970). Relation of shell form to life habits of the Bivalvia (Mollusca). Geological Society of America Memoirs.

[ref-117] Stanley SM (1972). Functional morphology and evolution of byssally attached bivalve mollusks. Journal of Paleontology.

[ref-118] Stanley SM (1981). Infaunal survival: alternative functions of shell ornamentation in the Bivalvia (Mollusca). Paleobiology.

[ref-119] Stanley SM, Trueman ER, Clarke MR (1988). Adaptive morphology of the shell in bivalves and gastropods. Form and function.

[ref-120] Stanley SM, Campbell LD (1981). Neogene mass extinction of Western Atlantic molluscs. Nature.

[ref-121] Stenzel HB (1964). Oysters: composition of the Larval Shell. Science.

[ref-122] Strotz LC, Saupe EE, Kimmig J, Lieberman BS (2018). Metabolic rates, climate and macroevolution: a case study using Neogene molluscs. Proceedings of the Royal Society B: Biological Sciences.

[ref-123] Sutherland C, Hare D, Johnson PJ, Linden DW, Montgomery RA, Droge E (2023). Practical advice on variable selection and reporting using Akaike information criterion. Proceedings of the Royal Society B: Biological Sciences.

[ref-124] Thomas CD, Cameron A, Green RE, Bakkenes M, Beaumont LJ, Collingham YC, Erasmus BFN, De Siqueira MF, Grainger A, Hannah L, Hughes L, Huntley B, Van Jaarsveld AS, Midgley GF, Miles L, Ortega-Huerta MA, Peterson AT, Phillips OL, Williams SE (2004). Extinction risk from climate change. Nature.

[ref-125] Tierney JE, Poulsen CJ, Montañez IP, Bhattacharya T, Feng R, Ford HL, Hönisch B, Inglis GN, Petersen SV, Sagoo N, Tabor CR, Thirumalai K, Zhu J, Burls NJ, Foster GL, Goddéris Y, Huber BT, Ivany LC, Turner SK, Lunt DJ, McElwain JC, Mills BJW, Otto-Bliesner BL, Ridgwell A, Zhang YG (2020). Past climates inform our future. Science.

[ref-126] Todd JA (2001). Introduction to molluscan life habits databases. Neogene Marine Biota of Tropical America (NMITA).

[ref-127] Todd JA, Jackson JBC, Johnson KG, Fortunato HM, Heitz A, Alvarez M, Jung P (2002). The ecology of extinction: molluscan feeding and faunal turnover in the Caribbean Neogene. Proceedings of the Royal Society of London B.

[ref-128] Troncoso OP, Torres FG, Arroyo J, Gonzales KN, Fernández-García M, López D (2020). Mechanical properties of calcite- and aragonite-based structures by nanoindentation tests. Bioinspired, Biomimetic and Nanobiomaterials.

[ref-129] Ubukata T (2005). Theoretical morphology of bivalve shell sculptures. Paleobiology.

[ref-130] Vermeij GJ (1987). Evolution and escalation: an ecological history of life.

[ref-131] Vermeij GJ (2007). The ecology of invasion: acquisition and loss of the siphonal canal in gastropods. Paleobiology.

[ref-132] Vermeij GJ (2017). Life in the arena: infaunal gastropods and the late Phanerozoic expansion of marine ecosystems into sand. Palaeontology.

[ref-133] Vermeij GJ (2020). Overcoming the constraints of spiral growth: the case of shell remodelling. Palaeontology.

[ref-134] Vermeij GJ (2022). The balanced life: evolution of ventral shell weighting in gastropods. Zoological Journal of the Linnean Society.

[ref-135] Violle C, Navas M-L, Vile D, Kazakou E, Fortunel C, Hummel I, Garnier E (2007). Let the concept of trait be functional!. Oikos.

[ref-136] Voigt E (1973). *Hydrallmania graptolithiformis* n. sp. eine durch Biomuration erhaltene Sertulariidae (Hydrozoa) aus der Maastrichter Tuffkreide. Paläontologische Zeitschrift.

[ref-137] Webster NB, Vermeij GJ (2017). The varix: evolution, distribution, and phylogenetic clumping of a repeated gastropod innovation. Zoological Journal of the Linnean Society.

[ref-138] White CR, Alton LA, Bywater CL, Lombardi EJ, Marshall DJ (2022). Metabolic scaling is the product of life-history optimization. Science.

[ref-139] White CR, Kearney MR (2013). Determinants of inter-specific variation in basal metabolic rate. Journal of Comparative Physiology B.

[ref-140] White CR, Marshall DJ (2023). How and why does metabolism scale with body mass?. Physiology.

[ref-141] Widdicombe S, Spicer JI, Gattuso J-P, Hansson L (2011). Effects of ocean acidification on sediment fauna. Ocean acidification.

[ref-142] Zubakov VA, Borzenkova II (1988). Pliocene palaeoclimates: past climates as possible analogues of mid-twenty-first century climate. Palaeogeography, Palaeoclimatology, Palaeoecology.

[ref-143] Zuur AF, Ieno EN, Elphick CS (2010). A protocol for data exploration to avoid common statistical problems. Methods in Ecology and Evolution.

