# Supplementary figures and images for: Differences in extinction selectivity and their relationship to functional traits in late Cenozoic mollusks
Source: PeerJ. 2026 Mar 3;14:e20715. doi: 10.7717/peerj.20715 (PMC12965174; doi:10.7717/peerj.20715)

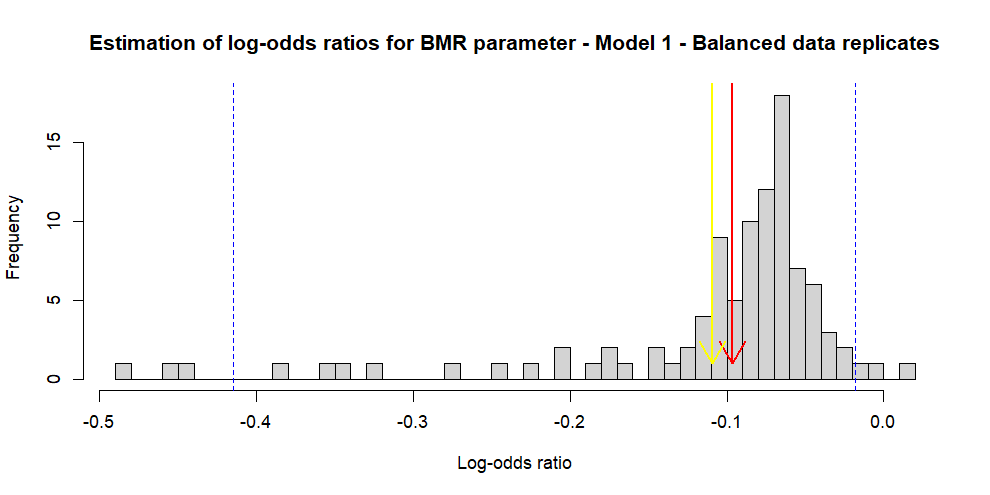

Supplement: Supplemental Information 1 — Estimation of effect size uncertainty via subsampling - 100 balanced replicates (38 bivalves - 38 gastropods). The red arrow indicates the true log-odds ratio, the yellow arrow indicates the mean log-odds ratio of the distribution of resampled estimates, and blue dotted lines indicate the 95% confidence interval of this distribution. [file peerj-14-20715-s001.png]

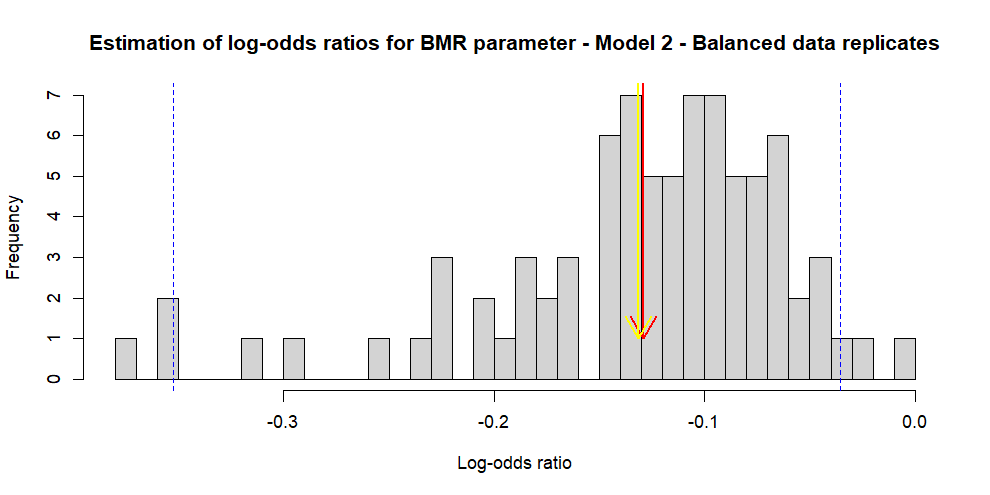

Supplement: Supplemental Information 2 — Estimation of effect size uncertainty via subsampling - 100 balanced replicates (38 bivalves - 38 gastropods). The red arrow indicates the true log-odds ratio, the yellow arrow indicates the mean log-odds ratio of the distribution of resampled estimates, and blue dotted lines indicate the 95% confidence interval of this distribution. [file peerj-14-20715-s002.png]
