# Supplementary material for: Differences in extinction selectivity and their relationship to functional traits in late Cenozoic mollusks
Source: PeerJ. 2026 Mar 3;14:e20715. doi: 10.7717/peerj.20715 (PMC12965174; doi:10.7717/peerj.20715)
Supplement: Supplemental Information 10 — BMR stands for basal metabolic rate. Chi-squared statistics and p-values for each trait were obtained from type III ANOVA tests performed on logistic regression models. Corrected Akaike Information Criterion scores (AICc) for each model are listed. Log-odds ratios are provided for each trait and for the intercept of each model. Intercepts represent the log-odds of survival when BMR is equal to 0 and/or the categorical traits of a model are set to their reference level. Interaction terms are indicated with an ‘x’, the reference level used for BMR was: ‘low’, the shell composition reference level used was: ‘aragonite’, and the reference level used for shell ornamentation was: ‘smooth ornament’. Levels of statistical significance are labeled as follows: * α < 0.05 and ** α < 0.01. Models that passed our selection criteria are highlighted in bold. [file peerj-14-20715-s010.docx]

| **Model** | **AICc** | **Trait** | **Chi-squared statistic** | **P-value** | **Logistic regression predictor** | **Log-odds ratio** |
| --- | --- | --- | --- | --- | --- | --- |
| **Shared traits** | | | | | | |
| **Model 1** | 148.0 | - | - | - | Intercept | -0.167 |
|  |  | BMR (categorized) | 5.90* | 0.049 | Medium BMR | 0.515 |
|  |  |  |  |  | High BMR | 0.410 |
| Model 2 | 149.2 | - | - | - | Intercept | -0.282 |
|  |  | Shell composition | 2.54 | 0.12 | Shell composition – Aragonite/low Mg calcite | 0.454 |
| **Shared traits – Bivalves only** | | | | | | |
| **Model 1** | 83.5 | - | - | - | Intercept | 0.178 |
|  |  | BMR (categorized) | 16.47** | 0.00 | Medium BMR | 1.429 |
|  |  |  |  |  | High BMR | 0.032 |
| Model 2 | 93.6 | - | - | - | Intercept | 0.064 |
|  |  | Shell composition | 4.02 | 0.06 | Shell composition – Aragonite/low Mg calcite | 0.577 |
| **Bivalves-specific traits** | | | | | | |
| **Model 1** | 83.46 | - | - | - | Intercept | 0.178 |
|  |  | BMR (categorized) | 16.47** | 0.00 | Medium BMR | 1.429 |
|  |  |  |  |  | High BMR | 0.362 |
| Model 2 | 87.71 | - | - | - | Intercept | 0.673 |
|  |  | Shell ornamentation | 12.22** | 0.00 | Shell ornamentation – fine ornament | 1.882 |
|  |  |  |  |  | Shell ornamentation – coarse ornament | -0.383 |
