# Supplementary material for: Differences in extinction selectivity and their relationship to functional traits in late Cenozoic mollusks
Source: PeerJ. 2026 Mar 3;14:e20715. doi: 10.7717/peerj.20715 (PMC12965174; doi:10.7717/peerj.20715)
Supplement: Supplemental Information 11 — BMR stands for basal metabolic rate. Chi-squared statistics and p-values for each trait were obtained from type III ANOVA tests performed on logistic regression models. Corrected Akaike Information Criterion scores (AICc) for each model are listed. Log-odds ratios are provided for each trait and for the intercept of each model. Intercepts represent the log-odds of survival when BMR is equal to 0. Levels of statistical significance are labeled as follows: * α ¡ 0.05 and ** α ¡ 0.01. Models that passed our selection criteria are highlighted in bold. [file peerj-14-20715-s011.docx]

| **Model** | **AICc** | **Trait** | **Chi-squared statistic** | **P-value** | **Logistic regression predictor** | **Log-odds ratio** |
| --- | --- | --- | --- | --- | --- | --- |
| **Shared traits** | | | | | | |
| **Model 1** | 144.9 | - | - | - | Intercept | -0.167 |
|  |  | BMR | 6.84* | 0.02 | BMR | -0.097 |
| **Shared traits – Bivalves only** | | | | | | |
| **Model 1** | 89.0 | - | - | - | Intercept | 0.787 |
|  |  | BMR | 8.63** | 0.00 | BMR | -0.110 |
