# Supplementary material for: Differences in extinction selectivity and their relationship to functional traits in late Cenozoic mollusks
Source: PeerJ. 2026 Mar 3;14:e20715. doi: 10.7717/peerj.20715 (PMC12965174; doi:10.7717/peerj.20715)
Supplement: Supplemental Information 12 — BMR stands for basal metabolic rate. Chi-squared statistics and p-values for each trait were obtained from type III ANOVA tests performed on logistic regression models. Corrected Akaike Information Criterion scores (AICc) for each model are listed. Log-odds ratios are provided for each trait and for the intercept of each model. Intercepts represent the log-odds of survival when BMR is equal to 0 and/or the categorical traits of a model are set to their reference level. Interaction terms are indicated with an ‘x’, the shell composition reference level used was: ‘aragonite’. Levels of statistical significance are labeled as follows: * α ¡ 0.05 and ** α ¡ 0.01. Models that passed our selection criteria are highlighted in bold. [file peerj-14-20715-s012.docx]

| **Model** | **AICc** | **Trait** | **Chi-squared statistic** | **P-value** | **Logistic regression predictor** | **Log-odds ratio** |
| --- | --- | --- | --- | --- | --- | --- |
| **Shared traits** | | | | | | |
| **Model 1** | 144.9 | - | - | - | Intercept | 0.253 |
|  |  | BMR | 6.84* | 0.02 | BMR | -0.097 |
| Model 2 | 143.8 | - | - | - | Intercept | 0.217 |
|  |  | Life habit | 10.05** | 0.008 | Life habit – semi-infaunal | 0.764 |
|  |  |  |  |  | Life habit – epifaunal | -0.083 |
| Model 3 | 149.2 | - | - | - | Intercept | -0.282 |
|  |  | Shell composition | 2.54 | 0.12 | Shell composition – Aragonite/low Mg calcite | 0.454 |
| Model 4 | 139.4 | - | - | - | Intercept | 0.496 |
|  |  | BMR | 6.61* | 0.17 | BMR | -0.086 |
|  |  | Life habit | 9.82* | 0.01 | Life habit – semi-infaunal | 0.864 |
|  |  |  |  |  | Life habit – epifaunal | -0.118 |
| Model 5 | 146.1 | - | - | - | Intercept | 0.022 |
|  |  | BMR | 5.28* | 0.03 | BMR | -0.086 |
|  |  | Shell composition | 0.98 | 0.34 | Shell composition – Aragonite/low Mg calcite | 0.295 |
| **Model 6** | 143.2 | - | - | - | Intercept | 0.365 |
|  |  | BMR | 9.54** | 0.007 | BMR | -0.169 |
|  |  | BMR x Life habit | 6.04 | 0.08 | BMR x Life habit – semi-infaunal | 0.128 |
|  |  |  |  |  | BMR x Life habit –epifaunal | -0.024 |
| **Model 7** | 144.2 | - | - | - | Intercept | 0.285 |
|  |  | BMR | 6.96* | 0.01 | BMR | -0.129 |
|  |  | BMR x Shell composition | 2.80 | 0.09 | BMR x Shell composition – Aragonite/low Mg calcite | 0.075 |
| Model 8 | 141.5 | - | - | - | Intercept | 0.499 |
|  |  | BMR | 6.28* | 0.02 | BMR | -0.092 |
|  |  | Life habit | 7.21 | 0.04 | Life habit – semi-infaunal | 0.805 |
|  |  |  |  |  | Life habit –epifaunal | -0.079 |
|  |  | BMR x Shell composition | 0.18 | 0.67 | BMR x Shell composition – Aragonite/low Mg calcite | 0.018S |
| Model 9 | 144.9 | - | - | - | Intercept | 0.377 |
|  |  | BMR | 9.84* | 0.01 | BMR | -0.185 |
|  |  | BMR x Life habit | 3.79 | 0.22 | BMR x Life habit – semi-infaunal | 0.228 |
|  |  |  |  |  | BMR x Life habit –epifaunal | -0.092 |
|  |  | BMR x Shell composition | 0.55 | 0.53 | BMR x Shell composition – Aragonite/low Mg calcite | -0.084 |
| **Shared traits – Bivalves only** | | | | | | |
| **Model 1** | 89.0 | - | - | - | Intercept | 0.787 |
|  |  | BMR | 8.63** | 0.006 | BMR | -0.110 |
| Model 2 | 93.6 | - | - | - | Intercept | 0.064 |
|  |  | Shell composition | 4.02 | 0.05 | Shell composition – Aragonite/low Mg calcite | 0.577 |
| Model 3 | 88.3 | - | - | - | Intercept | 0.624 |
|  |  | BMR | 6.98* | 0.02 | BMR | -0.097 |
|  |  | Life habit | 5.34 | 0.24 | Life habit – semi-infaunal | 0.780 |
|  |  |  |  |  | Life habit –epifaunal | -0.227 |
| **Model 4** | 89.8 | - | - | - | Intercept | 0.591 |
|  |  | BMR | 6.13* | 0.02 | BMR | -0.094 |
|  |  | Shell composition | 1.52 | 0.24 | Shell composition - Aragonite/low Mg calcite | 0.369 |
| Model 5 | 87.3 | - | - | - | Intercept | 1.014 |
|  |  | BMR | 11.71** | 0.007 | BMR | -0.181 |
|  |  | BMR x Life habit | 6.33 | 0.07 | BMR x Life habit – semi-infaunal | 0.132 |
|  |  |  |  |  | BMR x Life habit –epifaunal | -0.026 |
| **Model 6** | 88.1 | - | - | - | Intercept | 0.887 |
|  |  | BMR | 9.06** | 0.007 | BMR | -0.149 |
|  |  | BMR x Shell composition | 3.19 | 0.07 | BMR x Shell composition – Aragonite/low Mg calcite | 0.085 |
| Model 7 | 90.2 | - | - | - | Intercept | 0.686 |
|  |  | BMR | 7.03* | 0.02 | BMR | -0.113 |
|  |  | Life habit | 2.68 | 0.32 | Life habit – semi-infaunal | 0.639 |
|  |  |  |  |  | Life habit – epifaunal | -0.171 |
|  |  | BMR x Shell composition | 0.54 | 0.49 | BMR x Shell composition – Aragonite/low Mg calcite | 0.035 |
