# Supplementary material for: Differences in extinction selectivity and their relationship to functional traits in late Cenozoic mollusks
Source: PeerJ. 2026 Mar 3;14:e20715. doi: 10.7717/peerj.20715 (PMC12965174; doi:10.7717/peerj.20715)
Supplement: Supplemental Information 13 — Statistically significant associations of traits with survival are highlighted in bold. [file peerj-14-20715-s013.docx]

| **Trait 1** | **Trait 2** | | **Chi-squared p-value** | **Rank** | | | **Benjamin-Hochberg critical value** | | **Statistical significance** |
| --- | --- | --- | --- | --- | --- | --- | --- | --- | --- |
| Organism/substrate relationship | Mobility | 0.000 | | | 1 | 0.005 | | Significant | |
| Organism/substrate relationship | Shell fixation | 0.000 | | | 2 | 0.010 | | Significant | |
| Mobility | Shell fixation | 0.000 | | | 3 | 0.014 | | Significant | |
| **Status** | **Shell ornamentation** | **0.002** | | | **4** | **0.019** | | **Significant** | |
| Mobility | Shell ornamentation | 0.002 | | | 5 | 0.024 | | Significant | |
| **Status** | **Mobility** | **0.004** | | | **6** | **0.029** | | **Significant** | |
| Shell fixation | Shell ornamentation | 0.004 | | | 7 | 0.033 | | Significant | |
| Shell fixation | Feeding type | 0.005 | | | 8 | 0.038 | | Significant | |
| Feeding type | Shell ornamentation | 0.017 | | | 9 | 0.043 | | Significant | |
| **Status** | **Organism/substrate relationship** | **0.021** | | | **10** | **0.048** | | **Significant** | |
| Status | Feeding type | 0.057 | | | 11 | 0.052 | | Not significant | |
| Organism/substrate relationship | Feeding type | 0.064 | | | 12 | 0.057 | | Not significant | |
| Status | Shell fixation | 0.081 | | | 13 | 0.062 | | Not significant | |
| Feeding type | Ridges morphology | 0.084 | | | 14 | 0.067 | | Not significant | |
| Mobility | Feeding type | 0.093 | | | 15 | 0.071 | | Not significant | |
| Organism/substrate relationship | Shell ornamentation | 0.107 | | | 16 | 0.076 | | Not significant | |
| Shell fixation | Ridges morphology | 0.118 | | | 17 | 0.081 | | Not significant | |
| Status | Ridges morphology | 0.199 | | | 18 | 0.086 | | Not significant | |
| Organism/substrate relationship | Ridges morphology | 0.258 | | | 19 | 0.090 | | Not significant | |
| Ridges morphology | Shell ornamentation | 0.383 | | | 20 | 0.095 | | Not significant | |
| Mobility | Ridges morphology | 0.667 | | | 21 | 0.100 | | Not significant | |
