# Supplementary material for: Differences in extinction selectivity and their relationship to functional traits in late Cenozoic mollusks
Source: PeerJ. 2026 Mar 3;14:e20715. doi: 10.7717/peerj.20715 (PMC12965174; doi:10.7717/peerj.20715)
Supplement: Supplemental Information 14 [file peerj-14-20715-s014.docx]

| **Trait 1** | **Trait 2** | **Chi-squared p-value** | **Rank** | **Benjamin-Hochberg critical value** | **Statistical significance** |
| --- | --- | --- | --- | --- | --- |
| Feeding type | Umbilicus | 0.000 | 1 | 0.007 | Significant |
| Siphonal canal | Varices | 0.026 | 2 | 0.013 | Not significant |
| Status | Feeding type | 0.037 | 3 | 0.020 | Not significant |
| Siphonal canal | Umbilicus | 0.127 | 4 | 0.027 | Not significant |
| Feeding type | Siphonal canal | 0.237 | 5 | 0.033 | Not significant |
| Feeding type | Callus | 0.253 | 6 | 0.040 | Not significant |
| Varices | Callus | 0.263 | 7 | 0.047 | Not significant |
| Status | Siphonal canal | 0.401 | 8 | 0.053 | Not significant |
| Umbilicus | Callus | 0.412 | 9 | 0.060 | Not significant |
| Siphonal canal | Callus | 0.558 | 10 | 0.067 | Not significant |
| Status | Umbilicus | 0.763 | 11 | 0.073 | Not significant |
| Varices | Umbilicus | 0.791 | 12 | 0.080 | Not significant |
| Status | Varices | 0.828 | 13 | 0.087 | Not significant |
| Feeding type | Varices | 0.912 | 14 | 0.093 | Not significant |
| Status | Callus | 1 | 15 | 0.100 | Not significant |
