# Supplementary material for: Differences in extinction selectivity and their relationship to functional traits in late Cenozoic mollusks
Source: PeerJ. 2026 Mar 3;14:e20715. doi: 10.7717/peerj.20715 (PMC12965174; doi:10.7717/peerj.20715)
Supplement: Supplemental Information 15 — In this alternative dataset, predatory and browsing carnivores (CP and CB respectively in Table S3) were clumped into a single category of carnivores (C). Similarly, herbivores on fine-grained substrates (HM) and herbivores on plant or algal substrates (HP) were grouped into a single category of herbivores (H). [file peerj-14-20715-s015.docx]

| **Trait 1** | **Trait 2** | **Chi-squared p-value** | **Rank** | **Benjamin-Hochberg critical value** | **Statistical significance** |
| --- | --- | --- | --- | --- | --- |
| Siphonal canal | Varices | 0.024 | 1 | 0.007 | Not significant |
| Feeding type | Siphonal canal | 0.047 | 2 | 0.013 | Not significant |
| Siphonal canal | Umbilicus | 0.134 | 3 | 0.020 | Not significant |
| Feeding type | Umbilicus | 0.145 | 4 | 0.027 | Not significant |
| Status | Feeding type | 0.170 | 5 | 0.033 | Not significant |
| Varices | Callus | 0.229 | 6 | 0.040 | Not significant |
| Feeding type | Callus | 0.314 | 7 | 0.047 | Not significant |
| Status | Siphonal canal | 0.389 | 8 | 0.053 | Not significant |
| Umbilicus | Callus | 0.413 | 9 | 0.060 | Not significant |
| Siphonal canal | Callus | 0.588 | 10 | 0.067 | Not significant |
| Status | Umbilicus | 0.783 | 11 | 0.073 | Not significant |
| Varices | Umbilicus | 0.799 | 12 | 0.080 | Not significant |
| Status | Varices | 0.842 | 13 | 0.087 | Not significant |
| Feeding type | Varices | 0.893 | 14 | 0.093 | Not significant |
| Status | Callus | 1.000 | 15 | 0.100 | Not significant |
