# Supplementary material for: Differences in extinction selectivity and their relationship to functional traits in late Cenozoic mollusks
Source: PeerJ. 2026 Mar 3;14:e20715. doi: 10.7717/peerj.20715 (PMC12965174; doi:10.7717/peerj.20715)
Supplement: Supplemental Information 16 — In this alternative dataset, ridges morphologies and feeding types were reclassified. Ridges classified as “quasi-commarginal” in bivalves originally, were included in the “commarginal” category, and “quasi-radial” ridges were considered as “radial”. Regarding feeding type in this group, surface deposit feeders (labeled as DS in Table S2) and chemosymbiotic deposit feeders (DC in Table S2) were grouped into one category of deposit feeders (DF). Statistically significant associations of traits with survival are highlighted in bold. [file peerj-14-20715-s016.docx]

| **Trait 1** | **Trait 2** | **Chi-squared p-value** | | **Rank** | **Benjamin-Hochberg critical value** | **Statistical significance** |
| --- | --- | --- | --- | --- | --- | --- |
| Organism/substrate relationship | Mobility | 0.000 | 1 | | 0.005 | Significant |
| Organism/substrate relationship | Shell fixation | 0.000 | 2 | | 0.010 | Significant |
| Organism/substrate relationship | Feeding type | 0.000 | 3 | | 0.014 | Significant |
| Mobility | Shell fixation | 0.000 | 4 | | 0.019 | Significant |
| Shell fixation | Feeding type | 0.000 | 5 | | 0.024 | Significant |
| Mobility | Feeding type | 0.001 | 6 | | 0.029 | Significant |
| Mobility | Shell ornamentation | 0.002 | 7 | | 0.033 | Significant |
| Feeding type | Ridges morphology | 0.002 | 8 | | 0.038 | Significant |
| Shell fixation | Shell ornamentation | 0.003 | 9 | | 0.043 | Significant |
| **Status** | **Shell ornamentation** | **0.004** | **10** | | **0.048** | **Significant** |
| **Status** | **Mobility** | **0.005** | **11** | | **0.052** | **Significant** |
| **Status** | **Feeding type** | **0.016** | **12** | | **0.057** | **Significant** |
| **Status** | **Organism/substrate relationship** | **0.024** | **13** | | **0.062** | **Significant** |
| Feeding type | Shell ornamentation | 0.051 | 14 | | 0.067 | Significant |
| Status | Shell fixation | 0.093 | 15 | | 0.071 | Not significant |
| Organism/substrate relationship | Shell ornamentation | 0.113 | 16 | | 0.076 | Not significant |
| Status | Ridges morphology | 0.116 | 17 | | 0.081 | Not significant |
| Organism/substrate relationship | Ridges morphology | 0.198 | 18 | | 0.086 | Not significant |
| Shell fixation | Ridges morphology | 0.206 | 19 | | 0.090 | Not significant |
| Ridges morphology | Shell ornamentation | 0.351 | 20 | | 0.095 | Not significant |
| Mobility | Ridges morphology | 0.712 | 21 | | 0.100 | Not significant |
